# Megakaryocytes respond during sepsis and display innate immune cell behaviors

**DOI:** 10.3389/fimmu.2023.1083339

**Published:** 2023-03-02

**Authors:** Galit H. Frydman, Felix Ellett, Julianne Jorgensen, Anika L. Marand, Lawrence Zukerberg, Martin K. Selig, Shannon N. Tessier, Keith H. K. Wong, David Olaleye, Charles R. Vanderburg, James G. Fox, Ronald G. Tompkins, Daniel Irimia

**Affiliations:** ^1^ Division of Comparative Medicine and Department of Biological Engineering, Massachusetts Institute of Technology, Cambridge, MA, United States; ^2^ BioMEMS Resource Center and Center for Engineering in Medicine and Surgery, Department of Surgery, Massachusetts General Hospital, Boston, MA, United States; ^3^ Department of Pathology, Massachusetts General Hospital, Boston, MA, United States; ^4^ Harvard Neurodiscovery Center, Harvard Medical School, Boston, MA, United States

**Keywords:** megakaryocyte, platelet, sepsis, innate, infectious

## Abstract

Megakaryocytes (MKs) are precursors to platelets, the second most abundant cells in the peripheral circulation. However, while platelets are known to participate in immune responses and play significant functions during infections, the role of MKs within the immune system remains largely unexplored. Histological studies of sepsis patients identified increased nucleated CD61^+^ cells (MKs) in the lungs, and CD61^+^ staining (likely platelets within microthrombi) in the kidneys, which correlated with the development of organ dysfunction. Detailed imaging cytometry of peripheral blood from patients with sepsis found significantly higher MK counts, which we predict would likely be misclassified by automated hematology analyzers as leukocytes. Utilizing *in vitro* techniques, we show that both stem cell derived MKs (SC MKs) and cells from the human megakaryoblastic leukemia cell line, Meg-01, undergo chemotaxis, interact with bacteria, and are capable of releasing chromatin webs in response to various pathogenic stimuli. Together, our observations suggest that MK cells display some basic innate immune cell behaviors and may actively respond and play functional roles in the pathophysiology of sepsis.

## Introduction

Megakaryocytes (MKs) are commonly recognized as key participants in hemostatic processes through the production of platelets (1, 2). In addition to their presence in the bone marrow, MKs can also be located in the lungs, lymph nodes, spleen, and liver during extra medullary hematopoiesis (3–7). MKs have also been reported to be significantly increased in the lungs during severe pulmonary inflammation, such as acute respiratory distress syndrome (ARDS), where they are believed to promote inflammation *via* the local release of platelets (8–10). The current paradigm regarding higher numbers of MKs in the lungs during ARDS revolves around an increased passive escape rate of MKs from the bone marrow, likely facilitated by vasodilation or increased vascular permeability. This passive escape is proposed to then be followed by entrance into the arterial circulation and mechanical entrapment within the microcirculatory bed of the alveoli (4, 11, 12).

The participation of MKs in the immune response is suggested by several anecdotal observations (13): maturing MKs express both major histocompatibility complex (MHC) class I and II molecules and a variety of toll-like receptors (TLRs) on their cell surface (14–18); MKs, just like platelets, contain various granule types, including lysosomes, which participate in the endocytosis and degradation of pathogens (19); MKs can play antigen-presenting-cell (APC) roles and stimulate Th-17 responses in lupus (14, 20); thrombocytes, the amphibian equivalent of the mammalian MK/platelet, actively phagocytose live bacteria (21–23); and in mammals, MKs internalize and are infected by viruses, including dengue virus and HIV, with multiple case reports also showing evidence for MKs containing fungi (24–27). Although such reports provide sporadic evidence that MKs play an active role in immune responses, this aspect of MK function has not been tested systematically and is likely dependent on the type of pathogen or inflammatory signaling to which they are exposed.

Here, we found that during sepsis, the number of nucleated CD61^+^CD41^+^ cells was increased in the peripheral circulation, as was the number of large nucleated CD61^+^ cells in the pulmonary parenchyma. Strikingly, these numbers were amplified during acute kidney injury (AKI), ARDS, and in disseminated intravascular coagulation (DIC). As evidence that human MKs might actively respond to infection as innate immune cells rather than being passively released, we show that, *in vitro*, human MKs are capable of undergoing chemotaxis in response to gradients of standard chemo attractants, actively interact with pathogens, and may release extracellular chromatin webs in response to pathogenic stimulus.

## Results

### Platelets and megakaryocytes are increased in peripheral organs during sepsis

**Figure 1:**
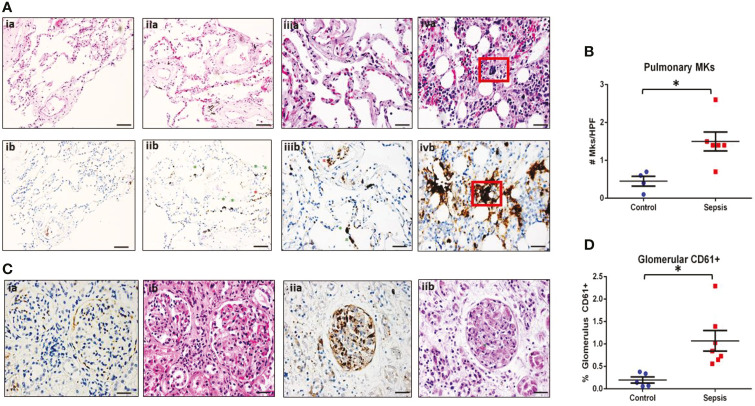
MKs are increased in peripheral organs during sepsis. Autopsy pathology samples from patients that died from sepsis were evaluated for MKs. **(A)** MKs in the lungs were defined as large cells with dark, homogenous CD61+ staining (brown). An example of a lung image from a patient that died from heart disease as a negative control is shown in panel ia (hematoxylin and eosin, H&E) and ib (CD61). No MKs were observed in these images. A representative image of two lung images from a patient that died from sepsis are shown in panel iia/iiia (H&E) and iib/iiib (CD61), showing multiple MKs (green asterisks). Platelet staining is also noted (red asterisks). In some cases, a large dark staining basophilic nucleus can be seen in the H&E in the area of the CD61 staining. This was not always the case, which may be due to sectioning and imaging of slightly different planes. This also suggests that CD61 may be more accurate when counting MKs than H&E. A sample of bone marrow from a sepsis patient is shown in panel iva (H&E) and ivb (CD61) as an example of an MK with CD61 staining as a positive control (red box). **(B)** Glomeruli were evaluated for the presence of increased CD61 staining. Individual MKs could not be reliable counted within the glomeruli, therefore percent of CD61 stain/glomeruli was evaluated. A glomerulus from a control patient is shown in panel ia (CD61) and ib (H&E), with minimal CD61 staining. This is in contrast to a glomerulus from a patient with sepsis and disseminated intravascular coagulation (DIC), which has much higher amounts of CD61 staining along with microvascular thrombi within the glomerular capillaries (green asterisk). Upon evaluation, there was significantly higher pulmonary MKs **(C)** and glomerular CD61 staining **(D)** in the sepsis patients as compared to control. Significance is calculated *via* student t-test with significance being defined as p < 0.05 (*). Scale bars are: Ai-ii, 100 μm, Aiii-iv and C, 50 μm.

We performed detailed histological analysis of autopsy samples from sepsis patients and non-sepsis controls to investigate whether platelets or MKs might be increased in the peripheral organs of patients with sepsis (**Table S1**). MKs were defined as CD61^+^ cells with large, multi-lobular, dark staining nuclei (as seen in **Figure 1A**). Per 40x field of view (FOV) of lung sections, large CD61^+^ MKs were significantly increased in the alveoli of septic patients (n = 8) as compared to controls (n = 4) (p = 0.01: 1.4 ± 0.2 MK/FOV vs 0.5 ± 0.1 MK/FOV, respectively) (**Figures 1A, B**). Comparing H&E-stained sections to corresponding CD61 IHC image showed that, although MKs could generally be identified on H&E by their large, darkly stained nucleus, this was not always the case. This demonstrates the importance of utilizing IHC for accurate cell-type assessment in these types of analysis (**Figures 1A, C**). Scoring of kidney sections revealed that renal glomeruli from septic patients (n = 7) also had a significantly increased amount of CD61+ positive staining than the controls (n = 5) (p = 0.018; 0.16 ± 0.08% CD61/Glomerulus vs 1.07 ± 0.23% CD61/Glomerulus, respectively) (**Figures 1C, D**). Comparing matched H&E and CD61 IHC glomeruli staining, it was not possible to differentiate with certainty whether large CD61^+^ areas corresponded to MKs or whether they represented platelet-rich fibrin thrombi (**Figure 1C**). One patient, who exhibited substantial microvascular thrombosis within the kidney and was diagnosed with disseminated intravascular coagulation (DIC), had significantly elevated CD61 staining (2.29 ± 3.0% CD61/Glomerulus), consistent with the presence of platelet-rich micro thrombi within the glomerular capillaries (**Figure 1D**).

### Megakaryocytes are increased in the peripheral venous circulation during sepsis

**Figure 2:**
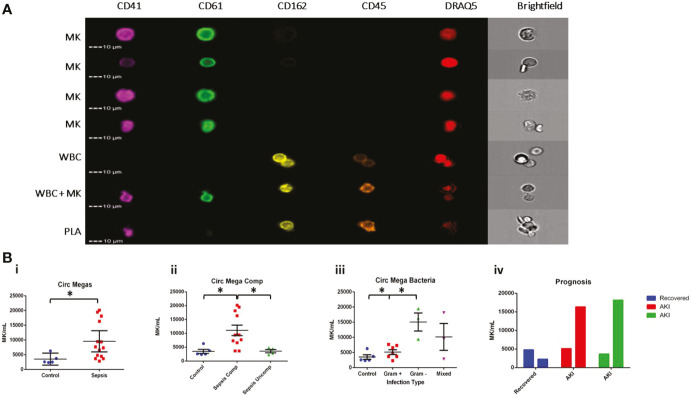
MKs are increased in the circulation during sepsis. Samples from patients diagnosed with sepsis were evaluated for the presence of possible MKs. MKs were identified in the peripheral circulation based on the simultaneous expression of CD41, CD61, and DRAQ5. **(A)** Imaging flow cytometry was used to identify and quantify circulating CD41+CD61+Draq5+ cells in the peripheral circulation. CD45 and CD162 were used as white blood cell markers. The top 4 panels show examples of MKs, while the bottom three panels show examples of two white blood cells, a white blood cell attached to a MK, and a white blood cell attached to a platelet (PLA). The cells negative for all markers in the images are likely red blood cells. **(B)** Circulating MKs were significantly higher in the peripheral circulation in patients with sepsis (i). The amount of MKs was correlated with sepsis-related complications, including ARDS and AKI, with ‘complicated’ sepsis having significantly higher MKs than ‘uncomplicated’ sepsis (ii). MKs were higher in gram negative and mixed infections compared to gram positive only infections (iii). Bar chart representing the MK count in 3 patients with sepsis that had blood collected on >1 day during the study. One patient recovered (blue) and two patients (red and green) developed AKI by day 3 of sample collection, suggesting that there also may be a correlation with recovery and the development of sepsis complications, such as AKI (iv). Statistics: student t-test **(Bi)** and ANOVA **(Bii, iii)** we performed, with significance defined as p < 0.05 (*). No statistical analysis was performed on the data in panel Biv due to the small sample size (n = 1 per group).

Since CD61^+^ MKs were increased in the peripheral organs of sepsis patients, we tested whether they could also be identified in the venous peripheral circulation. To maximize the specificity of our experiment, we employed imaging flow cytometry (AMNIS ImageStream) to probe for the presence of MKs in peripheral blood samples from patients. Circulating MKs were on average 10 µm in size and defined by the presence of CD61^+^ and CD41^+^ markers and Draq5^+^ staining of the nucleus. We differentiated MKs from platelet-leukocyte aggregates by visual inspection of the distribution of cell surface markers, which was uniform throughout the cell membrane for MK cells versus punctate when a platelet was attached to a leukocyte (**Figure 2A**). Use of imaging flow cytometry was imperative for the positive identification of circulating MKs, since there is high risk of false positive identification with clusters of platelets and nucleated cells. It is also important to note that unlike the culture-derived stem cell (SC) MKs and the Meg-01 cells utilized later, the peripheral circulating MKs identified in adult patients are CD162^-^. This suggests that there are different markers expressed by MKs depending on both the stage of maturation as well as the source of the cell, i.e. bone marrow, cord blood or peripheral circulation (28). Due to limitations on the number of co-stains for the imaging flow cytometry approach used, we did not include CD34 in the peripheral MK panel, so we cannot differentiate whether they are in the megakaryoblast or megakaryocyte stage.

The number of CD61^+^CD41^+^Draq5^+^ cells was significantly higher in the peripheral circulation of patients with sepsis compared to controls (p = 0.05; 9565 ± 1675 MK/mL vs 3502 ± 741 MK/mL, respectively) (**Figure 2Bi**). Interestingly, significant increases in MKs appeared to be specific to patients with ‘complicated’ (n = 9, including 2 follow-up counts on complicated patients) versus ‘uncomplicated’ sepsis (n = 4) and controls (n = 5) (p = 0.01 for t-test between complicated and uncomplicated or control; One-way ANOVA comparing all groups p = 0.0032; 11077 ± 6256 MK/mL vs 3587 ± 1219 MK/mL and 3502 ± 741 MK/mL, respectively) (**Figure 2Bii**), suggesting that there may be a correlation between the number of MKs in the peripheral venous circulation and the development of AKI or ARDS. When further subdividing the sepsis patient population by the source of the infection, patients with gram negative bacterial infections (n = 3) had significantly higher MK counts compared to those with gram positive bacterial infections (n = 7) (t-test p < 0.01) but not those with the mixed infections (n = 3) (t-test p = 0.13) (one way ANOVA comparing all groups, p = 0.001; 15070 ± 5158 MK/mL, 5140 ± 2082 MK/mL, 10146 ± 7675 MK/mL and controls 3502 ± 741 MK/mL respectively) (**Figure 2Biii**). Although the number of patients in this study was limited, the trend we observed suggests a possible link between the number of circulating megakaryocytes, the type of infection, and the clinical outcome.

### Automated analyzers fail to identify circulating megakaryocytes

We tested whether standard automated blood counting methods could identify circulating MKs (**Figure S1**). In an automated CBC analyzer, pure stem cell-derived MKs (SC MKs) (details in the Methods and Materials Section) were detected and categorized as an unknown type of white blood cells (WBCs) with an error notification. However, when SC MKs were spiked into whole blood samples, the automated analyzer categorized the additional cells as neutrophils, measured a corresponding increase in the WBC numbers, and did not display an error message (**Figure S1D**). Meg-01 cells could not be counted or analyzed accurately by the automated analyzer, either as a pure population or when spiked into whole blood. This is likely due to the differences in size between these two populations: While SC MKs are 10-30 µm and resemble small, granular lymphocytes on light microscopy, Meg-01 cells range from 15-75 µm and often form large clusters.

### Sequential sampling suggests increases in circulating megakaryocytes correlates with worse prognosis in sepsis patients

To further investigate whether MK enumeration in the circulation might be used to predict prognosis, we analyzed samples from three sepsis patients at 2 sequential time-points during their hospital stay (3 days apart). We observed a decrease in circulating MKs in the patient that was noted as recovering from sepsis on day 3 and was discharged from the hospital, as compared to the two patients that remained in the hospital for multiple weeks and developed AKI between day 1 and day 3 (**Figure 2Biv**). Platelet count did not significantly differ in sepsis versus control patients (p = 0.98; 251.1 ± 207.6 plt x 10^6^/mL and 253.6 ± 34.5 plt x 10^6^/mL, respectively), whereas there was a significant difference between sepsis and control patients in total white blood cell counts (p = 0.015; 14.7 ± 9.6 WBC x 10^6^/mL and 5.2 ± 1.1 WBC x 10^6^/mL, respectively). There was no correlation between circulating MKs and platelet or white blood cell count (**Figure S2**). While the sample size here is too small to draw definitive conclusions, our data suggest that circulating MKs should be further explored in the context of sepsis-related complications.

The observations above suggested that MKs are mobilized in response to infection, raising the possibility that they may play an active role in the immune response. To test this hypothesis, we performed a series of *in vitro* functional assays aimed at testing the innate immune activity of SC MKs and Meg-01 cells.

### Megakaryocytes undergo chemotaxis

**Figure 3:**
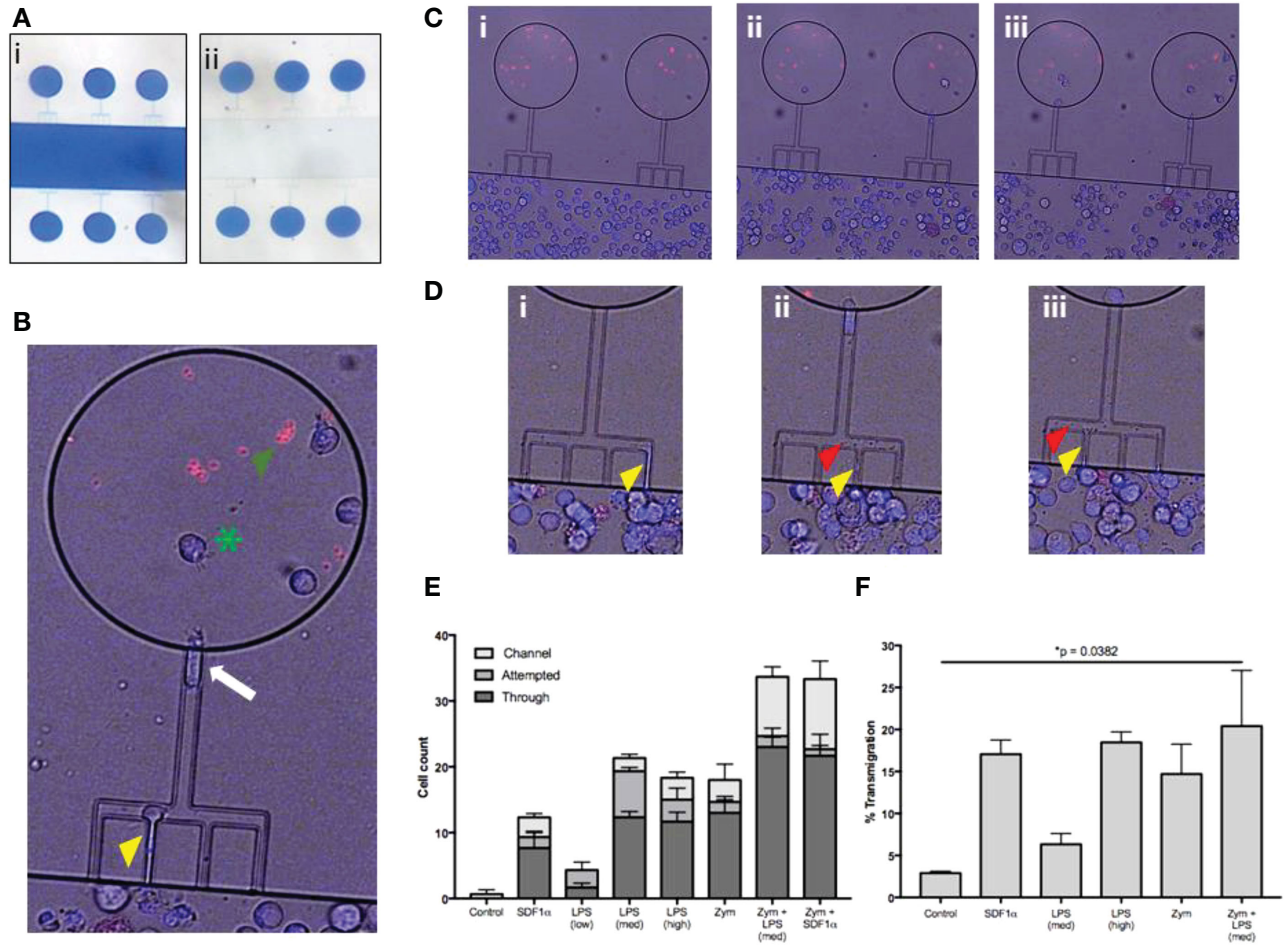
Meg-01 cells are capable of chemotaxis to pathogenic stimulus. Meg-01 cells were tested for their ability to chemotax towards LPS and zymosan particles. **(A)** A microfluidic device was used for part of the chemotaxis experiments. In this device, the main channel is connected to a circular reservoir by four 6 µm wide channels and a larger 8 µm wide connecting channel in a comb-like arrangement. The device is first primed with the condition (panel i) and the main channel is then flushed with media in order to create a concentration gradient from the chemoattractant chambers into the main channel (panel ii). **(B)** The MKs were stained with Hoechst for positive identification and then manually tracked. The behavior of the MKs was divided into 3 categories: cells attempting to enter the channel (yellow arrowhead), cells inside the channel (white arrow), and cells through the channel and inside the reservoir (green arrowhead). Zymosan particles are marked with a green asterisk. **(C)** Time lapse image of MKs migrating into chambers primed with LPS (360 pg/mL) and zymosan particles (**Ci**, no cells in the chambers with the red zymosan particles; **(Cii)**, cells starting to migrate up the combs into the zymosan chambers; **(Ciii)** final timepoint with multiple cells in each zymosan chamber). **(D)** Close-up of time-lapse imaging where MKs were observed attempting to enter the channel, extend a portion of the cell into the side channel (yellow arrowhead) **(Di–Diii)**, and then bud off small platelet-like particles (red arrowhead) **(Dii, Diii)**. Additionally, a single MK is followed shown throughout this timelapse first entering the comb **(Di)** and then finally entering the chamber **(Diii)**. **(E)** Bar graph representing MK chemotaxis within the microfluidic device. **(F)** Bar graph representing MK chemotaxis in a transwell assay, confirming the observations made with the microfluidic device. Statistical analysis for **(E, F)** are One-way ANOVAs with results in detail in **Figure S4**. LPS low, 22 pg/mL; LPS med, 220 pg/mL; LPS high, 2.2 ng/mL; Zym, zymosan particles; Zym+LPS, zymosan particles with 220 pg/mL LPS. Bar graphs are mean with standard error bars.

We tested the ability of Meg-01 cells to chemotax towards LPS and zymosan particles in microfluidic devices as well as traditional transwell assays (**Figure 3**). Meg-01 cells were used for the chemotaxis assays due to the high numbers of cells were required for robust assay performance with appropriate replicates. In microfluidic assays, we observed Meg-01 chemotaxis at single cell resolution and distinguished three phenotypic groups: cells in the first group migrated through the side channels and entered the chemoattractant circular reservoirs (“Through”); cells in the second group entered the side channels but did not reach the circular reservoir (“Channel”); and cells in the third group remained in the primary reservoir and extended projections into the side channels but did not fully enter the side channels (“Attempted”) (**Figures 3B, C**, **Figure S3**). An example of a Meg-01 cell migrating through these three stages can be found in **Video 1**. Strikingly, the size of the cells was not an impediment for cell migration, with large Meg-01 cells, up to 75 μm in diameter, actively migrated through 10.5 μm high and 4.5 μm wide (47.25 µm^2^ cross-section) channels. We observed nuclei and organelle displacement (**Videos 1, 2**), with some moving cells carrying zymosan particles within them (**Video 3**). A small proportion of cells did not fully traverse the channel, but instead extended portions into side channels containing chemoattractant. These projections released small cell fragments (platelets or apoptotic bodies) towards the stimulus (**Figure 3D**, **Video 4**). We also observed cells migrating into the side channels and then releasing platelets or vesicles (**Videos 5, 6**). Together, the increase in Meg-01 cell activity and chemotaxis behavior (“Through”) suggests MK cells are capable of actively responding to pathogenic stimulus, with statistically-significant increases in activity measured in all experimental conditions compared to controls, with the exception of low concentration LPS (22 pg/mL) and SDF1a (**Figures 3E**, **S3D**).

A traditional transwell migration experiment was also performed to verify the results from the novel microfluidic chemotaxis assay described. In the transwell experiments, the fraction of Meg-01 cells that migrated towards LPS at concentrations of 220 pg/mL and 2.2 ng/mL was 4.1-8.5% and 16.0-21.0%, respectively (**Figure 3F**). The fraction of cells migrating towards zymosan particles was comparable at 7.6-21.8% (**Figure 3F**). When LPS was combined with zymosan particles, the average chemotaxis fraction increased slightly compared to zymosan alone 7.1-33.7%, reaching statistical significance compared to the negative control (p = 0.0382) (**Figures 3F**, **S3D**). The positive control chemotaxis response of Meg-01 cells towards SDF1-α was between 13.7-20.4% (**Figure 3F**), consistent with previous reports (29).

### Megakaryocytes interact with but do not actively engulf bacteria

**Figure 4:**
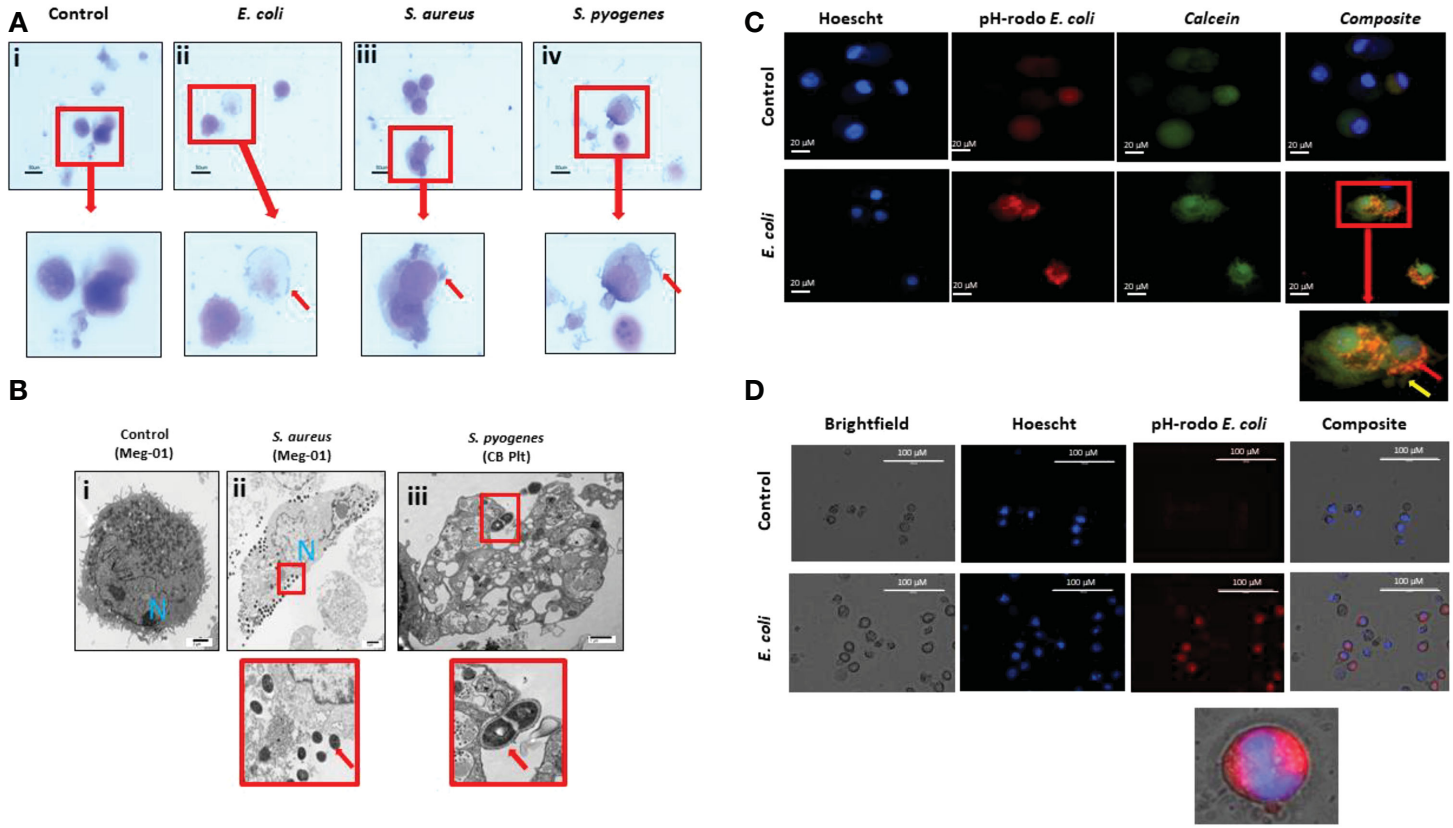
MKs interact with pathogens. **(A)** Meg-01 cells were co-incubated with *E. coli*, *S. aureus*, and *S. pyogenes*. Light microscopy with diff-quick staining shows that bacteria are associated with the cytoplasm and cell membrane of the cells. **(B)** Transmission electron microscopy of both Meg-01 and SC MKs exhibiting bacterial association with the cell membrane as well as internalization into vacuoles within the cytoplasm. Panel i is a control Meg-01 cell, while panel ii is a Meg-01 cell that was co-incubated with live *S. aureus.* Panel iii is from a SC MK culture co-incubated with live *S. pyogenes* showing association of the bacteria with a platelet cell membrane. **(C)** SC MKs were co-incubated with live pHrodo-conjugated bacteria. This is a representative image of a control cell along with a cell co-incubated with *E. coli*. **(D)** Meg-01 cells were co-incubated with pHrodo-conjugated live *E. coli* and then imaged. Notice the lack of rod-shapes in the Meg-01 cell **(D)** and diffuse cytoplasmic fluorescence in comparison with the SC MKs **(C)**. Bacteria, red arrow; pseudopods, yellow arrow.

Next, we tested whether MKs were capable of pathogen internalization and phagocytosis. We co-incubated SC MKs and Meg-01 cells with live *E. coli, S. aureus*, or *S. pyogenes* (**Figure 4**). Using light microscopy, we observed the frequent association of bacteria with the MK cell membrane (**Figure 4A**, **Video 7**). Using light microscopy, it was not possible to differentiate between bacteria that were internalized by the MKs versus bacteria that were merely bound to the periphery. Therefore, a series of advanced imaging experiments, including electron microscopy (EM) and fluorescently labeled bacteria and particles was utilized. Evaluation with transmission EM confirmed the frequent interaction between the bacteria and MKs and platelets (**Figure 4B**). Internalization of intact bacteria was rarely observed in MKs, with the bacteria most frequently appearing to localize around the periphery of the cell along the cell membrane or associating with intracellular contents that had been released into the extracellular compartment (**Figures 4A, Bii, Biv**). Interestingly, electron micrographs of a spontaneously contaminated SC MK culture at day 14 of differentiation revealed one large cell with multiple bacteria of unknown origin within an expansive vacuole (**Figure S5**). This spontaneous and incidental finding example clearly demonstrates pathogen internalization, although it is unclear whether the internalization observed was passive or active and whether it was initiated by the MK or the bacteria.

To characterize these interactions in more detail, we labeled pathogens with pHrodo, a marker that fluoresces red following acidification (presumably from interaction with the intracellular granule content). Cursory observation appeared to confirm the uptake of the bacteria within acidified compartments in the SC MKs (**Figure 4C**). SC MKs exhibited background auto fluorescence, however, bright rod-shaped *E. coli* were observed to be located diffusely around the cell with exclusion from the location of the nucleus, supporting the idea that these bacteria were internalized (otherwise the bacteria would have been observed to overlap the nucleus) (**Figure 4C**). Meg-01 cells were noted to exhibit very strong auto fluorescence (both red and green) and, at first, appeared to have internalized the pHrodo-labeled bacteria (**Figure 4D**). However, upon further investigation, with the use of use of dual-stained bacterial particles labeled with both Alexafluor-488 and pHrodo, it was confirmed that Meg-01 cells do not appear to internalize bacteria, but instead internalize any unbound pHrodo that may be present in the media. Further experiments utilizing human peripheral neutrophils as a positive control for bacterial particle phagocytosis and cytochalasin B (CCB) as an experimental phagocytosis inhibitor were performed (30, 31) (**Figure S6**). In this set of experiments, we confirmed that there was no phagocytosis of bacterial particles by Meg-01 cells (**Figure S6A**). Additionally, while CCB inhibited phagocytosis of the bacterial particles by the neutrophils, it appeared to enhance the internalization of the unbound pHrodo by the Meg-01 cells (**Figures S6B, C**).

At this time, it remains unclear whether this increase in red fluorescence is simply uptake of extracellular fluid containing the unbound pHrodo or if there are residual bacterial membrane fragments in the supernatant of the pHrodo-bacterial solution that can stimulate uptake by the cell (**Figure 4D**) *via* an alternative internalization mechanism, such as endocytosis or macropinocytosis, which has been demonstrated to occur in MKs and platelets (32–34). Additionally, while CCB is known to inhibit phagocytosis, it does not inhibit all forms of pinocytosis by cells such as macrophages (35, 36).

We performed time-lapse microscopy imaging of Meg-01 cells co-incubated with live pHrodo-stained *E. coli*. While not demonstrating active phagocytosis, this imaging exposed a complex and constant interaction between the Meg-01 cells and the bacteria. This interaction was characterized by the cells extending pseudopod-like structures in various directions upon co-incubation with both LPS and live bacteria, with frequent contact directly with the bacteria (**Video S7**). Interestingly, the bacteria appeared to consistently bind to one pole of the Meg-01 cell, resulting in a growing bacterial cluster and the opposite pole of the cell extending multiple protrusions, suggesting communication between the bacteria and the cells (**Video S8**).

### Megakaryocytes release chromatin webs

**Figure 5:**
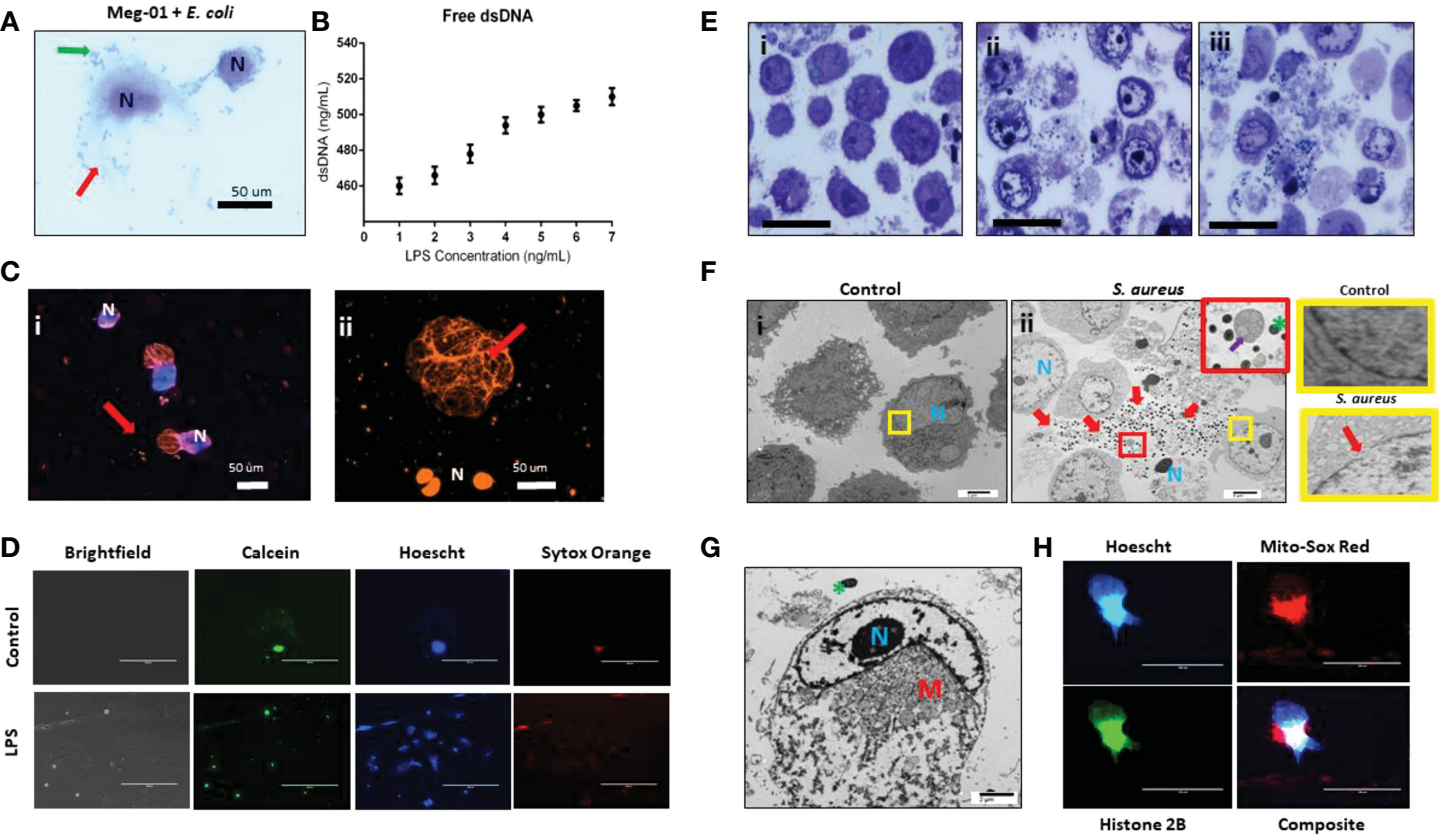
MK release chromatin webs. SC MKs and Meg-01 cells were observed to release chromatin webs in response to pathogenic stimulus. **(A)** Meg-01 cells co-incubated with live *E. coli* underwent cell lysis (diff-quick stain). Green arrow: bacteria; red arrow: extracellular cytoplasmic contents. **(B)** Chromatin released from Meg-01 cells after incubation with LPS quantified using PicoGreen assay. **(C)** SC MKs release chromatin webs after incubation with live pHrodo-conjugated *E. coli*. Live cell nuclei are blue (Hoechst stain). Chromatin webs are orange (Sytox stain). **(Bi)** SC MKS with nucleus (blue) and chromatin webs (orange). **(Bii)** SC MKs, one that has released a chromatin web (red arrow) and three dead cells with orange nuclei. **(D)** Meg-01 cells have intact cell membranes and proplatelet buddings (calcein staining). Cells incubated with LPS have scant calcein and abundant Sytox (extracellular chromatin) staining. **(E)** Meg-01 cells incubated with live bacteria display swollen nuclei, broken nuclear membranes, chromatin webs, extracellular granules, and bacteria associated with intra- and extracellular contents (light microscopy). **(Ei)** is the control, and **(Eii–iii)** are co-incubated with *E. coli* and *S. aureus*, respectively. **(Fi)** Transmission electron microscopy (TEM) of Meg-01 cells in media (control) revealed an intact nuclear membrane and limited extracellular content. Meg-01 cells co-incubated with live bacteria **(Fii)** display swollen nuclei (N), broken nuclear membranes, extracellular cytoplasmic contents (including granules and mitochondria), and an abundance of bacteria primarily associated with this extracellular content (red arrows). The red magnified section in **(Fii)** demonstrates the presence of extracellular mitochondria (purple arrow) and the yellow magnified sections on the right demonstrate an intact nuclear membrane in a control cell (top right) and a cell co-incubated with bacteria that has a break in the nuclear membrane (bottom right). **(G)** TEM image of a Meg-01 cell co-incubated with live *E. coli* exhibiting a swollen nucleus and a rearrangement of mitochondria surrounding the nucleus. **(H)** Meg-01 cells transfected with Bacmam H2b-GFP released chromatin webs that were both positive for DNA (Hoechst) and histone 2B. Mitochondrial staining with MitoSox red shows active mitochondria in a perinuclear arrangement, confirming the TEM findings from panel **(G)**.

We next explored how MKs would respond to challenge with various doses of pathogenic stimuli, specifically endotoxin. For this set of experiments, we used both Meg-01 cells and SC MKs at full maturation (culture day 14). Confirmation of differentiation into a mature SC MK and phenotyping of the Meg-01 cells was performed with imaging flow cytometry prior to the experiments (**Figures S7–S9**). We observed that Meg-01 cells incubated with live bacteria or LPS changed their cell morphology and released histone-decorated chromatin webs (**Figure 5**). Measurements of extracellular double stranded-DNA (dsDNA) in the supernatant *via* a PicoGreen assay demonstrated increases proportional to the concentration of LPS (**Figure 5B**). Fluorescent imaging of SC MKs co-incubated with live pHrodo-conjugated *E. coli* allowed us to visualize the chromatin webs and their filamentous structure (**Figure 5C**). During the early phase of chromatin web release, the nucleus of the cells was also stained by the Hoechst dye, which became more diffuse in the late stages, consistent with recent reports in the context of neutrophil extracellular trap (NETs) release (37). Immunofluorescent imaging confirmed the presence of extracellular histones and myeloperoxidase along with the chromatin webs (**Figure S10**). Although MPO has not been observed before in MKs using immunofluorescence techniques, positive MPO staining in our experiments along with the granular cytoplasmic staining pattern help to demonstrate the release of intracytoplasmic content alongside the release of extracellular chromatin. As previously reported, MKs contain histones within both their nucleus and their cytoplasm, therefore the presence of positive histone staining along the extracellular chromatin structure is likely from a combination of intranuclear and extranuclear histones (38).

Electron micrography of Meg-01 cells co-incubated with live bacteria confirmed the presence of bacteria entangled within the chromatin structures along with various organelles, including extracellular mitochondria, granules, and nucleosomes (**Figures 5E, F**). Fluorescence imaging using calcein staining to differentiate MK proplatelet formation from release of intranuclear contents in the form of extracellular chromatin webs; for proplatelet formation, calcein should be seen around any nuclear staining, whereas for extracellular chromatin release there would be nuclear staining without the associated calcein stain, signaling release of the intracellular contents and cell death. (**Figure 5D**). Other cell morphology changes consistent with both cell lysis and extracellular chromatin web formation included swollen nuclei, breakdown of the nuclear membrane, decondensed chromatin, and re-localization of the cytoplasmic organelles and mitochondria (39, 40). Furthermore, Meg-01 cells expressing GFP-H2B were imaged actively releasing their intracellular contents, which confirmed the perinuclear rearrangement of organelles (notably, mitochondria) (**Figures 5G, H**).

## Discussion

**Figure 6:**
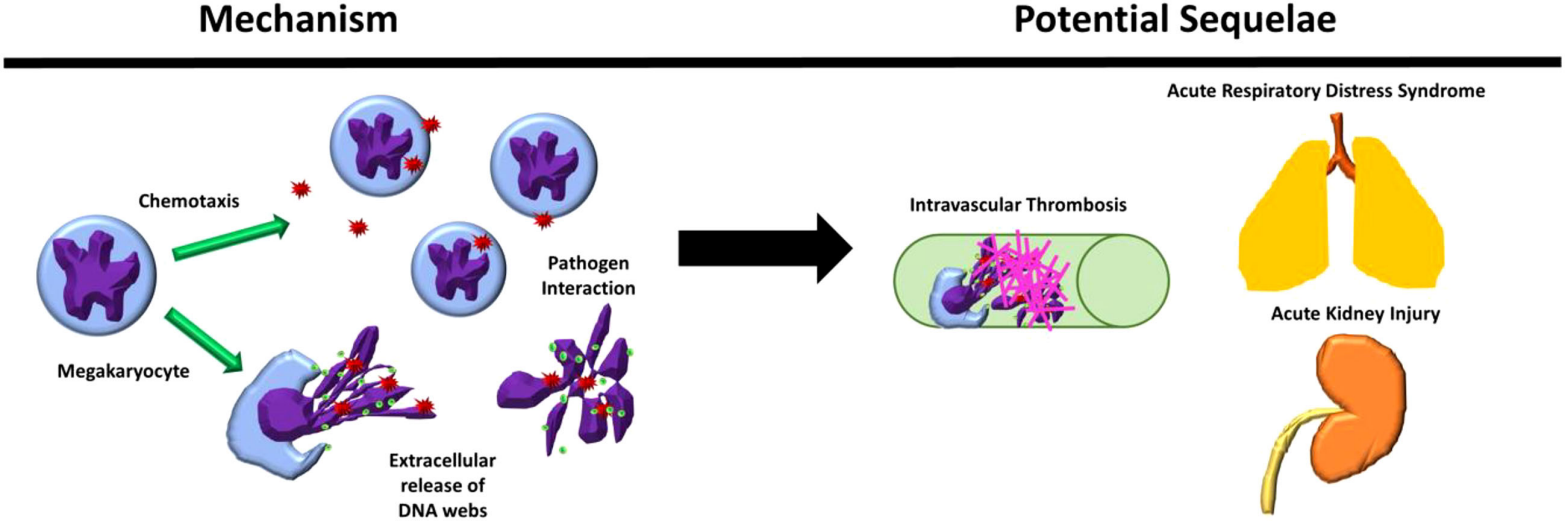
Megakaryocyte immune behaviors observed and their possible sequelae in physiological systems. Megakaryocytes (MKS) were observed to chemotax and release extracellular chromatin webs in response to pathogenic stimuli and interact directly with pathogens. These behaviors, in combination with their presence in increased amounts in the blood and peripheral organs during severe inflammatory conditions, such as sepsis, may contribute to the response to pathogenic stimulus, including the contribution to the development of intravascular thrombosis in peripheral organs, such as the lungs and the kidneys.

In this study, we identified increased MKs in the circulation and peripheral organs of patients with sepsis. These observations led us to explore the potential of MKs to function as innate immune cells, exposing them to various types of bacteria and bacterial toxin (endotoxin). We demonstrate that MK cells may display several immune cell functions, including chemotaxis, pathogen interaction, and the release of histone-decorated chromatin webs, in addition to their traditional role in thrombopoiesis (**Figure 6**) (2).

The potential role of MKs in pathology is especially important in the context of our finding that large numbers of MKs are present in the peripheral circulation and peripheral organs of patients with sepsis. These findings are consistent with previous reports showing that MK numbers are increased in the peripheral circulation in neonates with sepsis, in the renal glomeruli of adults with sepsis, and in the lungs in patients with sepsis and acute respiratory distress syndrome (ARDS) (41, 42). Peripheral MKs likely arise from multiple sources, including release from sites of extra medullary hematopoiesis, such as the liver and lymph node (frequently increased during inflammation), as well as the bone marrow compartment, due to increased vascular permeability and vasodilation during systemic inflammation and sepsis (41). In this study, it was not possible to identify the origin of the MKs found in the peripheral circulation. Regardless of the source of the MKs, the role of the MK in the immune response and inflammation should be considered, especially when located outside of their traditional niche.

When studying the peripheral circulation, special staining is usually required to identify and quantify MKs. Here we have demonstrated that automated hematology analyzers not only fail to identify MKs in the blood but can actually misclassify them as leukocytes (41, 43, 44). To positively identify circulating MKs, a protocol using imaging flow cytometry was required to visually differentiate MKs from platelet clusters or platelet-leukocyte aggregates (45–47). Like lymphocytes, the observed average circulating MK diameter was between 10-15 μm and exhibited scant cytoplasm. MKs identified in the lungs are typically larger in size than those described here found in the peripheral venous circulation, which may support the idea that these populations of larger cells become entrapped within the first microcirculatory bed they encounter upon their release into the circulation (48, 49). Alternatively, MKs in places such as the lungs could start off as larger in size, then upon activation and the release of platelets, be subsequently released into the circulation as smaller MKs due to scant cytoplasm.

We found that that Meg-01 cells undergo active chemotaxis towards common inflammatory stimuli (29) and can migrate through microchannels. Our observation raises the possibility that peripheral MKs ‘trapped’ in microcirculatory beds may intentionally move throughout and may even diapedese into surrounding tissues where they could participate in inflammation and regeneration (3, 50–54). Additionally, the observed directional budding of platelets into channels containing chemotactic agents and “obstruction” of the channels by MKs and platelets and/or vesicles suggest an ability to contribute to decreased blood flow within small capillary vessels through either physical obstruction or *via* stimulation of immunothrombosis (10, 55–59). These findings are especially interesting in the context of MKs located outside of the bone marrow niche, e.g., within the pulmonary parenchyma. Although it is likely that the MKs localize to the alveolar capillaries due to size constraints, this being the first microcapillary bed encountered upon release from the bone marrow, it is possible that once within the lungs, the MKs may be able to undergo chemotaxis and interact with pathogenic stimulants.

With this in mind, we also explored whether MKs interact with and phagocytose bacteria. While MKs appeared to interact with bacteria, which frequently bind to their cell membrane, they were rarely observed to internalize the bacteria and phagocytosis could not be confirmed as the mechanism of internalization in our experiments. These results suggest that internalization of pathogens may occur *via* an alternative mechanism, such as bacterial invasion of the MK host cell (58–61). Our finding complements earlier observations of MKs containing various intracellular pathogens, including dengue virus, human immunodeficiency virus, and aspergillus (24–27). In the case of viral infection, the pathogen enters the cell and then utilizes the MK as means of dissemination, releasing viral particles through their platelet progeny (26, 62). Our findings also align with the previous conflicting reports of the mechanism of pathogen internalization by platelets (63). We observed that MKs readily take up unbound pHrodo from their culture media, suggesting an alternate mechanism suggesting they may employ an alternative approach to active phagocytosis for pathogen sensing and antigen presentation, although the mechanism of internalization requires further investigation.

Interestingly, we observed that MKs are capable of responding to soluble pathogenic stimuli by releasing intracellular contents and generate chromatin webs. While it is expected for cells to undergo apoptosis or necrosis upon exposure to pathogenic stimuli such as high concentrations of LPS, certain types of cell death, such as extracellular trap formation by neutrophils (NETosis), are known to be highly pro-inflammatory, pro-thrombotic and bactericidal. This type of cell death involves the controlled release of intracellular contents and DNA, and is increasingly recognized to be shared with broad range of innate immune cells, including neutrophils, eosinophils, and monocytes/macrophages (40, 59). While in this study we did not perform experiments to confirm that the MK chromatin web release is directly comparable to NET formation, it does appear to contain many of the same characteristics as the traditional NETs, including the inclusion of extracellular DNA, histones and MK-related granular contents. Although this MK extracellular chromatin structure may not represent a classic extracellular trap (they lack positive staining for CitH3, a hallmark of NET formation) it is very likely that the MK-derived webs are highly proinflammatory and prothrombotic, similar to other apoptotic and necrosing cells. We suspect that these MK webs may also participate in disease processes, such as ARDs and sepsis, when there are increased numbers of MKs within the pulmonary parenchyma along with commonly observed localized microthrombi (57, 64, 65). If MKs localized within the pulmonary parenchyma are exposed to pathogens and inflammatory signals, this may in turn stimulate chemotaxis, platelet production, and/or extracellular DNA web release.

The role of the MK in the host immune response requires further study. Platelets are known to be ‘first responders’, stimulating the recruitment and activation of white blood cells, and even directly trapping pathogens themselves, which suggests that the parent MK may be capable of the same behavior (55, 66, 67). It is also important to note that platelets have recently been shown to present antigens in the context of MHC Class I (68). In this study, we demonstrate that MKs express MHC Class I (HLA-ABC) (**Figure S7**) throughout differentiation, so it is plausible that the interaction and potential internalization of the bacteria could result in MKs acting as antigen presenting cells (APCs) during infection, perhaps directly activating naïve T cells. A role for MKs as APCs has been suggested by previous studies. However, additional studies need to be conducted to further characterize the bacteria-MK interaction and any potential resultant leukocyte interaction/stimulation (20, 69), whether it is through direct MK interaction or indirectly through their platelet progeny.

It is also important to recognize the limitations of this exploratory study, which includes the use of the Meg-01 cell line and SC-derived MKs for the *in vitro* experiments. It is well known that depending on the source of CD34^+^ cells, the MKs derived from these source cells have significant variations in morphology, and therefore likely also have differences in cellular phenotype with relation to surface marker expression and behavior (70). Another limitation of this paper includes the lack of *in vivo* studies. Further in-depth characterization studies evaluating the role of MKs in the peripheral circulation and within organ parenchyma is essential. Using newly available techniques, such as intravital microscopy (4), it may now be possible to explore megakaryocyte behavior during different pathological conditions, such as mouse models of pneumonia or ARDS. Additionally, further studies evaluating peripheral MKs in various patient populations should also be performed in much larger cohorts and at various stages of sepsis.

Historically, the presence of MKs outside the bone marrow has been mostly ignored, suspected to be the result of either a hematopoietic cancer driving increased megakaryopoiesis, increased extra medullary hematopoiesis, or due to changes in vascular permeability resulting in MK release from the bone marrow. While the experimental findings presented in this paper may give rise to more questions than they may answer, they also serve to support a hypothesis where peripheral MK cells, rather than merely representing an artefact of disease, may functionally contribute to local inflammatory and/or coagulation changes, whether through direct interaction with pathogens or local stimulation and release of platelet progeny or intracellular contents.

## Materials and methods

### Pathology samples

Histopathology samples from patients that underwent autopsies were retrospectively collected and evaluated. All samples and patient information were collected and handled according to MGH and Massachusetts Institute of Technology (Cambridge, MA, USA) IRB protocol (MGH No: 2014P002087; MIT No: 150100681R001). A patient was categorized as septic when the cause of death was determined to be sepsis by the official pathology report. Control patients were defined as having primary cardiac disease as the cause of death. Fifteen samples were evaluated with 9 sepsis samples (age 60-90 yrs., 7 females, 2 males) and 5 control samples (age 68-87 yrs., 2 females, 3 males) (**Table S1** ). All histopathology slides were de-identified and analyzed blindly.

Paraffin embedded tissue samples, including kidney, and the right middle lung lobe were sectioned and stained with either Hematoxylin & Eosin (H&E), gram stain, or with HRP-labeled CD61 antibodies by the Division of Comparative Medicine (MIT, Cambridge, MA, USA) and the Massachusetts General Hospital (Boston, MA, USA), respectively. The percent of CD61 staining per renal glomerulus was quantified using ImageJ software (NIH). Twenty renal glomeruli were evaluated for each patient. For evaluation of the lungs, the number of MKs were counted in ten 40x magnification views of the right middle lung lobe for each patient.

### MK quantification in patient blood samples

Discarded venous blood, collected in Ethylenediaminetetraacetic acid (EDTA), from patients diagnosed with sepsis was collected and evaluated for circulating MKs in accordance with the protocol approved by the Partners Human Research Committee (PHRC) (IRB protocol numbers, MGH No: 2014P002087; MIT No: 150100681R001). All methods were carried out in accordance with relevant guidelines and regulations. Informed patient consent was waived by the ethics committee because of the use of discarded blood samples followed by de-identification. A patient was categorized as septic when one of the diagnoses for the patient was ‘sepsis’ and when there was a confirmed infection, which could consist of bacterial infection, fungal infection, viral infection, or a combination thereof. Control samples were healthy donors. ‘Complicated’ sepsis was defined as the clinician-diagnosed development of acute kidney injury (AKI) or acute respiratory distress syndrome (ARDS) as documented in the patient’s medical records. Twenty-one total samples were evaluated from 13 patients with sepsis (age 34-75 yrs., 4 females, 9 males), three of the sepsis samples (1 female and 2 males) had follow-up blood evaluation 3 days after initial blood analysis, and 5 control patients (age 25-50 yrs., 3 females, 1 male) (**Tables S2**, **S3**).

Cell surface markers were selected to determine cell type by differential marker expression. CD162 antibody was utilized to break up any platelet-leukocyte aggregates prior to analyzing the samples (45). White blood cell concentration was used as an internal control for the quantification method and whole blood spiked with MKs was used to validate surface marker identification of MKs in samples (**Figure S1**). Cell surface markers were selected to determine cell type by differential marker expression: CD41 (glycoprotein IIb; GPIIb) and CD61 (glycoprotein IIIa; GPIIIa) are found on the cell surface of platelets and MKs, where they form a GPIIb/IIIa complex and bind to fibrinogen and von Willebrand Factor (vWF) during platelet activation. CD45 (protein tyrosine phosphatase, receptor type, C; PTPRC; leukocyte common antigen; LCA) and CD162 (P-selectin glycoprotein ligand-1; PSGL1; SELPLG) are both present on leukocytes where CD45 hi and lo populations can be used to identify both neutrophils and lymphocytes. Draq5 is a nuclear marker that was used to differentiate between CD41^+^CD61^+^ anuclear platelets and nucleated MKs.

First, white blood cells were identified and quantified and were then compared to the total white blood cell count in the complete blood cell count (CBC) that was performed on the same blood sample at the Massachusetts General Hospital clinical pathology lab as part of the patient’s routine diagnostics to verify our concentration calculation methods (**Figures S1A–C**). Once white blood cell count was verified, cellular events staining positive for MK markers were then collected and quantified. The concentrations of MKs and leukocytes in the patient samples were then back-calculated, taking into account the total volume of sample analyzed and the initial 1:200 dilution of the blood.

To quantify circulating MKs in peripheral venous samples, we performed quantitative imaging flow cytometry on whole blood. In this set of experiments, we used the quantification of leukocytes in the blood as an internal methods control, with the quantity of leukocytes being compared to the automated CBC analyzer total white blood cell count to validate the cell concentration calculations. Blood collected in EDTA vacutainer tubes was diluted in calcium-free hepes-tyrode buffer (Boston Scientific, Boston, MA, USA) with 20% volume of acid citrate dextrose (ACD, Boston Scientific) (6 uL whole blood + 194 uL buffer). The diluted blood was then stained with CD41 PacBlue, CD61 FITC, CD45 CY5/594, and CD162 PE at 1:100-1:200 for 20 minute, and 1:1000 Draq5 for 5 minutes. The samples were then run using the Amnis flow cytometer and the data was analyzed with IDEAS software. MKs were defined as CD41^+^CD61^+^Draq5^+^ cells. Leukocytes were defined as CD162^+^CD45^+^Draq5^+^ cells. It is imperative to use imaging flow cytometry for the identification of circulating MKs, otherwise there may be false positive results such as with large platelet clumps around another type of nucleated cell. Each circulating MK that was identified in the patient samples using imaging flow cytometry was individually visually inspected to confirm the presence of a single nucleated cell with only MK-specific cell surface markers. The concentrations of MKs and leukocytes in the patient samples were then extrapolated, taking into account the total volume of sample analyzed by Amnis and the initial dilution of the blood (Equation 1).

**Equation 1:** (Cell count (# cells)/Total volume analyzed (uL)) x (dilution factor; 33.3) x (1000 uL/mL) = # MK/mL

To explore the ability of the automated CBC analyzer to count and identify MKs, venous blood collected in EDTA was spiked with various concentrations of Meg-01 cells or cord-blood derived MKs (day 14 of differentiation). Pure MKs and the spiked whole blood samples were analyzed with flow cytometry as described above, and also run on an automated CBC analyzer to determine whether an automated analyzer was able to detect the presence of MKs and to specify what type of cell they categorize as. Pure MKs were counted manually with a hemocytometer to compare with the automated analyzer (**Figures S1D, E**). While the imaging flow cytometer was able to specifically identify cells as MKs, the automated CBC analyzer was unable to identify them.

### Cell culture

Purified cord blood (CB) CD34^+^ hematopoietic stem cells were purchased from StemCell Technologies and cultured in StemSpan II media with the MK supplemental cytokines (StemSpan II, Stemcell Technologies, Inc.), according to the culture and differentiation protocols from Stemcell Technologies (Stemcell Technologies Inc. Cambridge, MA). These CD34^+^-derived MKs are referred to as SC-MKs throughout the paper. A megakaryoblastic cell line (Meg-01) was purchased from ATCC (American Type Culture Collection, Manassas, VA) and cultured in RPMI with 10% FBS, according to ATCC’s standard culture protocols. Meg-01 cells were used for experiments for both optimization of protocols prior to repetition with the SC CD34 cell line or when a high concentration of cells was needed for multiple replicates, such as the chemotaxis assays. Because Meg-01 cells are from a megakaryoblastic cell line and therefore may not exhibit the same behavior or receptors as the fully-mature, non-eternal MK cells, CD34^+^-derived MK cells were used as an additional MK model to further support our findings, although these cells may also not display the same behaviors as circulating MKs (48, 49).

### Flow cytometry

Flow cytometry was performed in order to verify appropriate cellular differentiation and for the evaluation of cell surface markers. Briefly, cells were stained with antibodies at a concentration of 1:200 for 15 minutes, with the exception of CD41, which was at a concentration of 1:100. Cells were then stained with Draq5 (Thermo Fisher Scientific, Waltham, MA) at a concentration of 1:10000 for 5 minutes. Antibodies included: anti-human CD41, HLA-ABC (MHC class I), HLA-DR (MHC class II), CD162 (p-glycoprotein-1; SELPG), CD61 (GPIIIa), CD41 (GPIIb), CD34 (Biolegend, San Diego, Ca), CD66b, and CD62P (P-selectin) (BD Biosciences, San Jose, CA). Data was obtained through the Amnis ImageStreamX Mark II imaging flow cytometer and INSPIRE Software (EMD Millipore, Billerica, MA). The accompanying IDEAS Software was used to perform data analysis. Data is reported as the percent of the total cell population that stained positive for the specific marker. Briefly, the rationale for the marker choices are as follows: CD34 represents the hematopoietic stem cell (HSC); CD41 and CD61 is an early marker of MK differentiation, and vWF is a marker of the promegakayoblast stage; HLA-ABC and HLA-DR were used to identify the presence of these immune-receptors during MK differentiation; CD162 is commonly present on immune cells was used to explore the expression of this marker on the maturing MK; CD62P is a platelet activation marker; and CD66b was used to identify granulocytes. Draq5 was used as a nuclear marker (**Figure S1B**).

### Transwell migration assay

Growth factor reduced (GFR) matrigel coated transwell inserts with 8 µm pores (Biocoat Matrigel Invasion Chambers, Corning, New Jersey, USA) were thawed for 30 minutes at 37^0^C. 1 million Meg-01 cells were loaded in a total volume of 200 µL of RPMI with 10% FBS in the top chamber, while the bottom chamber was loaded with 600 µL of various conditions, including: RPMI with 10% FBS (negative control), 200 ng/mL SDF1-α (CXCL12, Peprotech, New Jersey, USA) (positive control), 220 pg/mL and 2.2 ng/mL *E. coli* LPS, Zymosan particles alone, and zymosan particles with 220 ng/mL *E. coli* LPS or with 200 ng/mL SDF1-α. The wells were incubated for 24 hrs. at 37°C. The cells in that migrated through the transwell insert into the bottom chamber were counted using Cellometer Vision automated cell counter (Niexcelom, Bioscience LLC., Lawrence, MA, USA).

### Microfluidic device fabrication

The microfluidic devices were manufactured using standard microfabrication techniques. The microfluidic device was designed to allow the formation of a chemical gradient in two steps, as previously described (71). Briefly, a two-layer photoresist design (SU8, Microchem, Newton, MA), with a first and second layer that were 10.5 and 50 μm thick, were patterned on one silicon wafer *via* sequential photolithography masks and processing cycles according to the manufacturer’s protocols. The resulting patterned wafer was then used as a mold to produce PDMS (Polymidemethylsiloxane, Fished Scientific, Fair Lawn, NJ) devices, which were subsequently irreversibly bonded to glass slides (1x3 inches, Fisher). First, an array of circular wells (200 µm diameter, 57 µm height), connected to a side channel (10 µm width, 10.5 µm height) by orthogonal side-combs (4.5 µm width, 10.5 µm height) were primed with the following conditions: RPMI with 10% FBS (negative control), 200 ng/mL SDF1-α (CXCL12, Peprotech, New Jersey, USA) (positive control), 22 pg/mL, 220 pg/mL and 2.2 ng/mL *E. coli* LPS, zymosan particles, zymosan particles (1x10^6^/mL) with 220 pg/mL *E. coli* LPS and zymosan particles (1x10^6^/mL) with 200 ng/mL SDF1-α. 200 µL RPMI +10% FBS was used to wash the main channel. The diffusion of the chemoattractant from the circular wells, serving as sources, to the central channel, serving as the sink, produced the guiding gradient for the cells in the central channels. After the devices were primed and loaded, the chip was placed under vacuum for 10 minutes. Cells were then stained, loaded, and imaged for 18 hrs every 10 minutes using time-lapse imaging on a fully automated Nikon TiE microscope with the biochamber at 37^°^C and 80% humidity, and in the presence of 5% carbon dioxide gas. Images were acquired automatically from distinct locations on each microfluidic device, with each image including a minimum of 3 circular wells. A minimum of 18 wells per condition were analyzed. Fiji manual tracking software (NIH) was used for the analysis of MK and platelet migration and behavior.

### Bacterial conjugation to pHrodo

Bacteria, including *Escherichia coli (E. coli), Staphylococcus aureus (S. aureus), and Streptococcus pyogenes (S. pyogenes)*, were provided by the Division of Comparative Medicine at the Massachusetts Institute of Technology (Cambridge, MA). Bacteria were then conjugated to pHrodo succinimidyl ester dye (Thermo Fisher Scientific) according to the manufacturer’s protocols. Briefly, bacteria were pelleted from cultures by centrifugations at 5100 rpm for 10 minutes. Pellets were resuspended in PBS (pH 9.0) at a concentration of 1x10^8^ per mL. 200 µL of this suspension was then added to 5 µL of 10 mg/mL pHrodo succinimidyl ester dye and mixed thoroughly by pipetting. Bacteria were then stained for 30 mins in the dark with gentle shaking. Following staining, 1 mL of PBS (pH 8.0) was added to the solution, and bacteria were pelleted at 13,400 rpm for 3 minutes in a benchtop centrifuge. The supernatant was removed and the pellet thoroughly resuspended in Tris Buffer (pH 8.5). The bacteria were then pelleted at 13,400 rpm for 3 mins in a benchtop centrifuge, the supernatant was removed, and the bacteria were resuspended in 1 mL of PBS (pH 7.4) before they were stored at 4°C in the dark. Unfortunately, the final concentration of bacteria used in the phagocytosis experiments was not calculated.

### Bacterial phagocytosis inhibition assay

*Beta-hemolytic E. coli* was provided by the Division of Comparative Medicine at the Massachusetts Institute of Technology (Cambridge, MA). Bacteria were heat-killed by incubation at 98°C for 30 mins. Unlabeled *Staphylococcus aureus* (Wood strain without protein A) BioParticles were purchased from Thermo Fisher Scientific. Bacteria and BioParticles were pelleted by centrifugations at 5100 rpm for 10 minutes at 4°C in a swing bucket centrifuge. Pellets were resuspended in PBS (pH 9.0) at a concentration of 1x10^8 per mL. 200 µL of this suspension was then added to 5 µL of 10 mg/mL pHrodo Red succinimidyl ester dye +/- Alexa Fluor 488 succinimidyl ester dye (Life Technologies) and mixed thoroughly by pipetting. Bacteria were then stained for 30 mins in the dark with gentle shaking. Following staining, 1mL of PBS (pH 8.0) was added to the solution, and bacteria were pelleted at 13,400 rpm for 3 mins in a benchtop centrifuge. The supernatant was removed and the pellet was thoroughly resuspended in Tris Buffer (pH 8.5). Again, the bacteria were pelleted at 13,400 rpm for 3 mins in a benchtop centrifuge, the supernatant was removed, and the bacteria resuspended in 1 mL of PBS (pH 7.4).

Blood was collected in ACD Vacutainer tubes (BD, Becton, Dickinson and Company, Franklin Lakes, NJ). Neutrophils were isolated from whole blood using a negative-selection protocol. Briefly, neutrophils were isolated using a density gradient with HetaSep (STEMCELL Technologies Inc. Vancouver, Canada) and then purified with EasySep Human Neutrophil Kit (STEMCELL Technologies Inc. Vancouver, Canada), following manufacturers protocol. Neutrophil purity was assessed to be >98% and cell count was performed using a hemocytometer. Neutrophils were subsequently re-suspended in the same media as the Meg-01 cells (RPMI + 10% FBS).

Meg-01 and isolated neutrophils were incubated with 10 µg/mL Cytochalasin B from Dreschslera dematodia (C6762; Sigma-Aldrich) or DMSO for 30 mins prior to addition of bacteria. Cells were incubated for 2 hours with heat-killed bacteria co-labeled with pHrodo Red and Alex Fluor 488 to measure phagocytosis. 10 µL of cells were then imaged on a disposable C-Chip hemocytometer (In Cyto, SKC, Inc. C-Chip) using a 10X and 20X objective on a Nikon TiE fluorescent microscope. Stitched 6 x 6 field of view large images were de-identified and 100 cells scored blind for red fluorescence for the positive indication of pHrodo internalization and acidification. The following conditions were included in the experimental design and analysis: cells (negative control), cells with unstained bacteria (control), cells with CSC (control), cells with stained bacteria (experimental), cells with stained bacteria and CSC (experimental). No nucleated cells were noted to have a red fluorescent cytoplasm when not co-incubated with the stained bacteria. There was also no increase in the number of nucleated cells with red cytoplasm in the conditions only treated with CCB.

### Immunofluorescence imaging

Cells were co-incubated with either the pHrodo-conjugated bacteria, Zymosan A, or *S. cerevisiae* BioParticles (ThermoFisher Scientific), for 60 minutes or overnight at 37°C on poly-lysine coated slides (Sigma Aldrich, St Louis, MO, USA). The slides were then rinsed gently three times with PBS and the adhered cells were subsequently stained for live imaging or fixed and then stained. For evaluation of live cells, cells were stained with Hoechst (Thermo Fisher Scientific) at a concentration of 1:2000 for 5 minutes. For the calcein green-stained cells, cells were also stained with calcein (Thermo Fisher Scientific) at 1:1000 for 5 minutes. For chromatin web evaluation, cells were also stained with SYTOX orange (Thermo Fisher Scientific) at 1:50 for 5 minutes. For histone staining of extracellular contents, cells co-incubated with *E. coli* LPS for 60 minutes at 37°C. They were then fixed with 4% paraformaldehyde (Santa Cruz Biotechnology, Dallas, TX, USA) for 30 minutes and then concentrated onto a polylysine coated slide using the Cytospin 4 cytocentrifuge (Thermo Fisher Scientific), for 5 minutes at 1250 rpm, rinsed once with di-water and stored at -80°C until staining and evaluation. For staining, the slides were thawed at room temperature and blocked with 5% donkey serum (Jackson Immunoresearch) for 2 hours. The slides were rinsed three times with PBS and then treated with the following primary antibodies for three hours: mouse anti-human neutrophil elastase (NE; ELA2) antibody (950334, Novus Biologicals, Littleton, CO, USA) at 1:300, rat anti-Histone H3 (phospho S28) antibody (HTA28, Abcam, Cambridge, MA, USA) at 1:500, and rabbit anti-human myeloperoxidase (A039829-2, Dako, USA) at 1:300. The slides were then rinsed three times with PBS and incubated with secondary antibodies, including donkey anti-rabbit 488, donkey anti-rat 647, and donkey anti-mouse 568 (Life Technologies) at 1:500 for 30 minutes. Slides were then rinsed three times with PBS and then covered with Vectashield antifade mounting medium with DAPI (Vector Labs, Burlingame, Ca, USA). The cells were then images with one of two fluorescent microscopes: Life Technologies EVOS FL (Thermo Fisher Scientific) or Nikon Eclipse 90i microscope (Nikon Instruments Inc., Melville, NY). Composites and videos were made, and images were analyzed using either Fiji or GIMP software.

### Chromatin web release

Meg-01 and SC MK cells underwent various treatments to induce the formation of chromatin webs from MK cells. Meg-01 cells were co-incubated with various pathogenic stimuli, including *E. coli* LPS and live pHrodo conjugated *E. coli*, for 30-60 minutes at 37°C on a polylysine-coated slide. While the concentration of the bacteria used was not quantified for these experiments, LPS concentrations used ranged from 200 ng/mL-1 ug/mL. The polylysine slide was outlined with a hydrophobic pen prior to the experiment to set a boundary for the liquid. The slide was then rinsed three times in PBS. The slides were stained with 1:1000 Hoechst and 1:500 SYTOX orange and cover slipped. For the GFP-H2B experiments, Meg-01 cells were transfected with CellLight Histone 2B-GFP (Bacmam 2.0, ThermoFisher Scientific) for 48-72 hours and then labeled with MitoSox Red mitochondrial superoxide indicator (ThermoFisher Scientific) and Hoechst. Unstained slides were stored and evaluated with immunofluorescence techniques, as described above. The cells were imaged using one of two fluorescent microscopes: Life Technologies EVOS FL (Thermo Fisher Scientific) or Nikon Eclipse 90i microscope (Nikon Instruments Inc., Melville, NY).

### Double stranded-DNA (ds-DNA) quantification

Meg-01 cells, at 3 x 10^5^ per mL, were co-incubated with various concentrations of *E. coli* LPS (20 pg/mL to 2 ug/mL) for 30 minutes at 37°C. The cells were then pelleted down at 1900 g for 10 minutes and the supernatant was immediately stored at -80°C. The experiment was performed in biological triplicates (cells from 3 different culture flasks) and in technical triplicates, on two different days. Double stranded DNA (dsDNA) was quantified using the Quant-iT™ PicoGreen™ dsDNA Assay Kit (Thermo Fisher Scientific) following the recommended protocol. Briefly, the supernatant samples were thawed and 50 uL was placed in a 96-well plate, followed by 50 uL of the aqueous working solution. A standard curve was created for a reference of extracellular chromatin. The plate was incubated at room temperature for 5 minutes and then read at standard fluorescein wavelengths (excitation ~480 nm, emission ~520 nm) on a SpectraMax Gemini XS plate reader (Molecular Devices, Sunnyvale, CA, USA). The mean fluorescent intensities (MFIs) were then converted to free dsDNA concentration according to the standard curve.

### Transmission electron microscopy

Meg-01 cells were co-incubated with live bacteria for 1 hour at 37°C. The cells were then pelleted down at 1900 g for 10 minutes. Immediately after removal of the culture medium, KII fixative (2.5% glutaraldehyde, 2.0% paraformaldehyde, 0.025% Calcium Chloride in a 0.1M Sodium Cacodylate buffer, pH 7.4) was added to the cell/bacteria pellet, mixed, and allowed to fix for 20 minutes. The fixed sample was then prepared for both transmission electron microscopy and thin-section light microscopy and were subsequently imaged. Briefly, a rubber tipped cell scraper was used to gently remove the fixed monolayer from the plastic substrate. The samples were centrifuged, the fixative removed, replaced with buffer, and stored at 4°C until further processing. To make a cell block, the material was centrifuged again and resuspended in warm 2% agar in a warm water bath to keep the agar fluid. The material was then centrifuged again, and the agar allowed to gel in an ice water bath. The tissue containing tip of the centrifuge tube was cut off resulting in an agar block with the material embedded within it. This agar block was then processed routinely for electron microscopy in a Leica Lynx™ automatic tissue processor. Subsequent processing was done using a Leica Lynx™ automatic tissue processor. Briefly, they were post-fixed in osmium tetroxide, stained En Bloc with uranyl acetate, dehydrated in graded ethanol solutions, infiltrated with propylene oxide/Epon mixtures, embedded in pure Epon, and polymerized overnight at 60°C. One-micron sections were cut, stained with toluidine blue, and examined by light microscopy. Representative areas were chosen for electron microscopic study and the Epon blocks were trimmed accordingly. Thin sections were cut with an LKB 8801 ultramicrotome and diamond knife, stained with lead citrate, and examined in a FEI Morgagni transmission electron microscope. Images were captured with an AMT (Advanced Microscopy Techniques) 2K digital camera.

### Statistical analysis

Statistics were performed using both Microsoft Excel and GraphPad Prism Software (GraphPad Software, Inc.). Either one-way ANOVA or student t-tests were performed to compare between conditions. A p-value of <0.05 was considered significant.

## Data availability statement

The original contributions presented in the study are included in the article/**Supplementary Material**. Further inquiries can be directed to the corresponding authors.

## Ethics statement

The studies involving human participants were reviewed and approved by Mass General Brigham Human Research Committee. Written informed consent for participation was not required for this study in accordance with the national legislation and the institutional requirements.

## Author contributions

GF developed the hypothesis, designed and performed experiments, and wrote the manuscript. FE provided guidance on bacterial phagocytosis experiments and chemotaxis assays, performed bacterial assays and contributed to manuscript preparation. ST contributed to the flow cytometry experiments and assay analysis. LZ provided guidance and pathology review of the sepsis samples, contributed to manuscript preparation. MS Performed electron microscopy, contributed to manuscript preparation. JJ and AM maintained cell cultures and performed chemotaxis and phagocytosis experiments and data analysis. KW provided guidance on immunofluorescence experiments and contributed to manuscript preparation. DO performed automated image analysis on the kidney pathology samples. CV, DI, JF, and RT designed experiments, analyzed the findings, and prepared the manuscript. All authors contributed to the article and approved the submitted version.
