# Supplementary material for: Megakaryocytes respond during sepsis and display innate immune cell behaviors
Source: Front Immunol. 2023 Mar 2;14:1083339. doi: 10.3389/fimmu.2023.1083339 (PMC10019826; doi:10.3389/fimmu.2023.1083339)
Supplement: Supplementary file 1 [file DataSheet_1.docx]

**Supplementary information**

**Megakaryocytes Display Innate Immune Cell Behaviors and Respond during Sepsis**

Galit H. Frydman^1, 2, †*^, Felix Ellett^2, †^, Julianne Jorgensen^2^, Anika L. Marand^2^, Lawrence Zukerberg^3^, Martin Selig^3^, Shannon Tessier^2^, Keith H. K. Wong^2^, David Olaleye^1^, Charles R. Vanderburg^4^, James G. Fox^1^, Ronald G. Tompkins^2^, and Daniel Irimia^2 *^

^1^Division of Comparative Medicine and Department of Biological Engineering, Massachusetts Institute of Technology, Cambridge, Massachusetts, United States of America.

^2^BioMEMS Resource Center and Center for Surgery, Innovation & and Bioengineering, Department of Surgery, Massachusetts General Hospital, Boston, Massachusetts, United States of America.

^3^Department of Pathology, Massachusetts General Hospital, Boston, Massachusetts, United States of America.

^4^Harvard Neurodiscovery Center. Harvard Medical School. Boston, Massachusetts, United States of America.

Supplemental Tables 1 to 3

Supplemental Figures 1 to 11

Supplemental Videos 1 to 8

**Supplemental Table S1. Patient descriptive statistics for pathology analysis
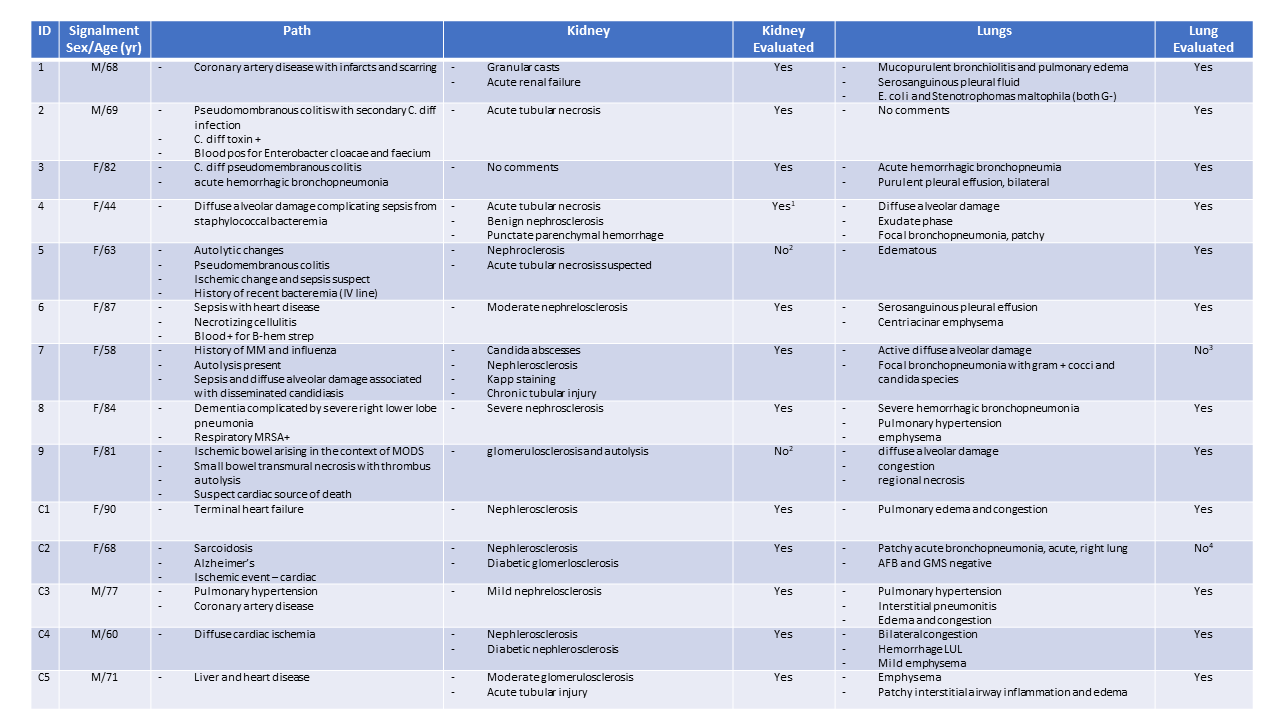
**

C, Control Patient; 1, DIC resulted in organ hemorrhage; 2, autolysis noted, staining too faint to analyze; 3, sample not available for analysis; 4, Sarcoidosis pathology in lungs.

**Supplemental Table S2. Patient descriptive statistics for circulating MKs**

**
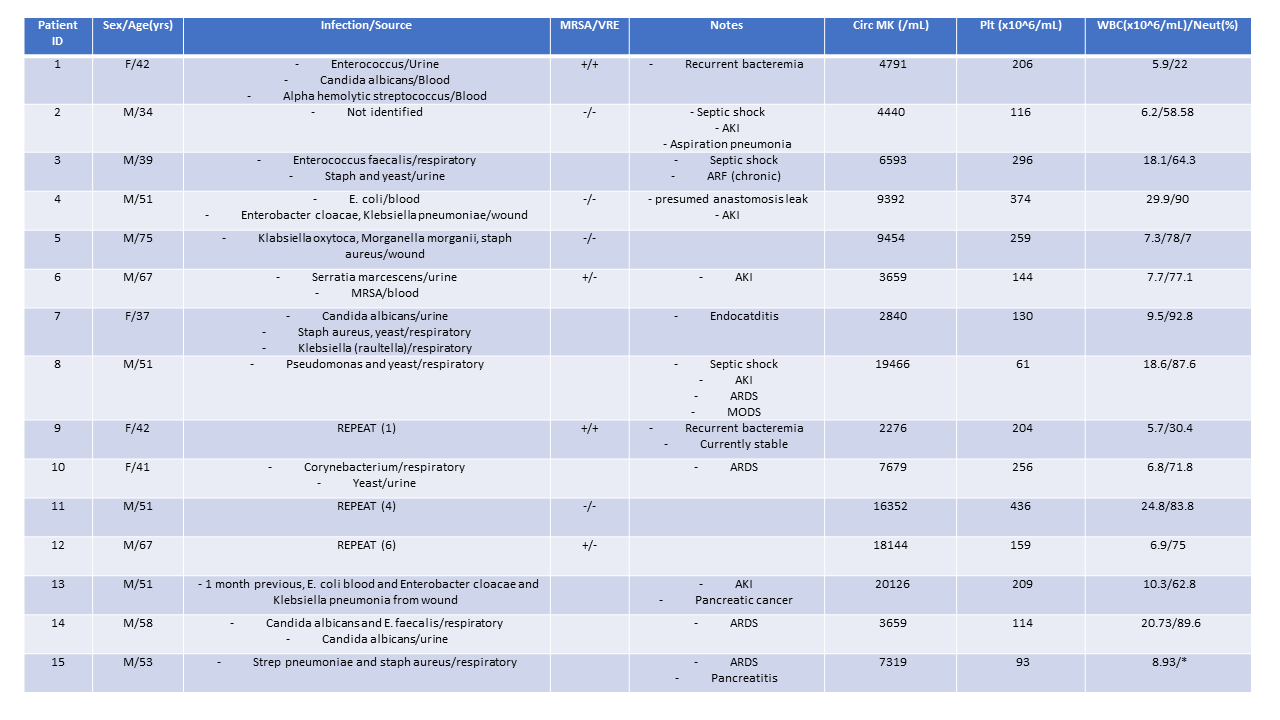
**

**Supplemental Table S3. Patient flow cytometry data for circulating MKs**

**
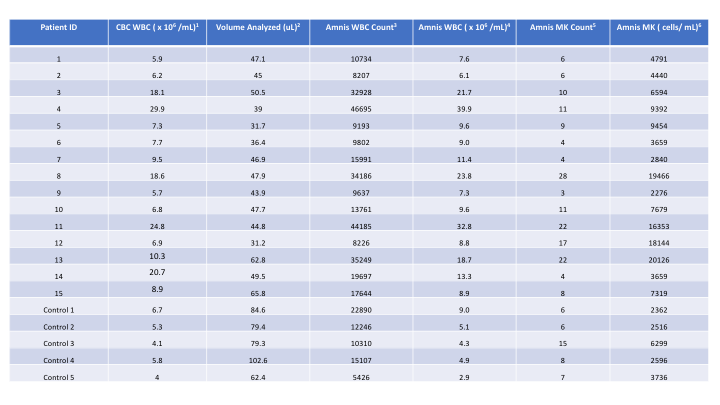
**

1, Complete Blood Cell Count from automated analyzer performed at the MGH Clinical Core Laboratory; 2, Total sample volume analyzed using Amnis flow cytometry. This sample is at a 1:33.3 dilution; 3, CD45+ hi and lo, CD162+ and DRAQ5+ cells; 4, Equation for WBC Count:((# WBC) / Volume (uL))*33.3) / 1000; 5, CD41+CD61+ and DRAQ5+ cells; 6, Equation for MK Count: (# MK / Volume (uL)) * 33.3 * 1000.

**
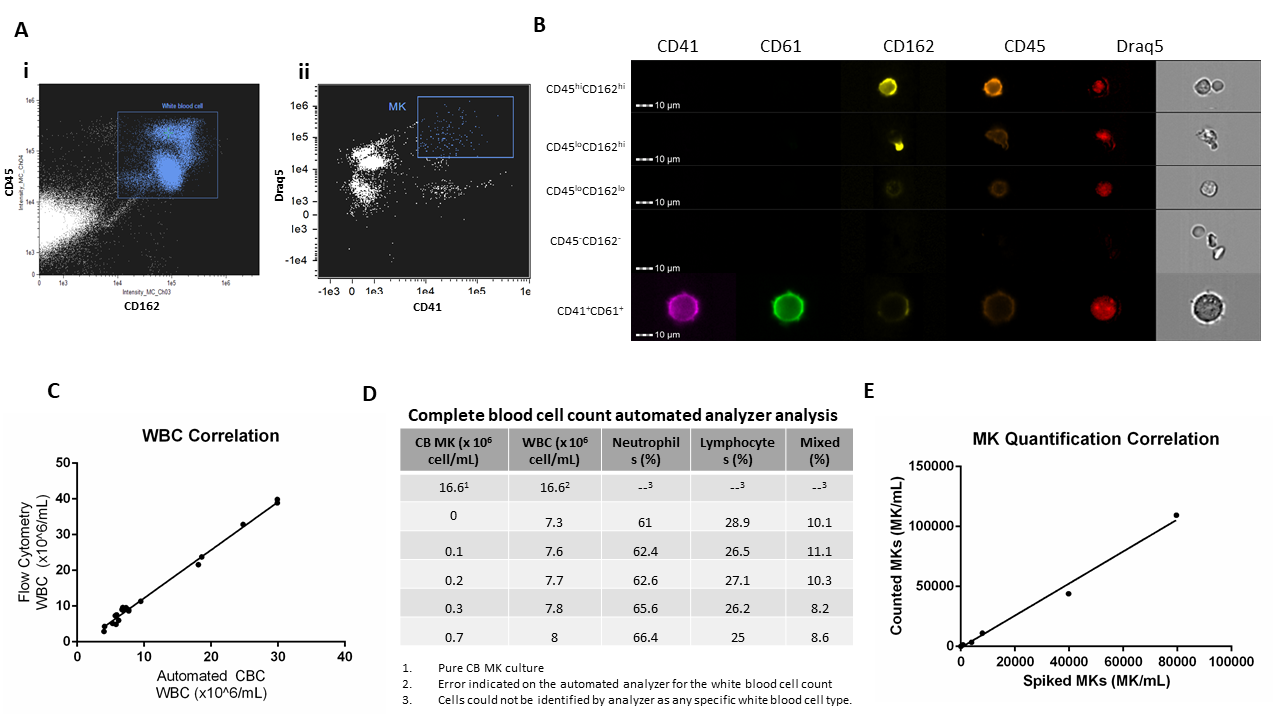
**

**Figure S1: Quantification of MKs in peripheral blood using flow cytometry.**

The positive identification and quantification of MKs in whole blood samples was evaluated. (A & B) A flow cytometry protocol was optimized for the accurate quantification of the total white blood cell population in whole blood. White blood cells were identified by being double positive for CD162 and CD45 (i). Meg-01 cells were identified by being positive for Draq5 and CD41 +/- CD61 (ii). Imaging flow cytometry demonstrates that CD45 and CD162 hi and lo populations were present, representing different types of white blood cells. The cells that were double negative for CD45 and CD162 were either platelets or red blood cells, and the large cells that were positive for CD41 and/or CD61 were Meg-01 cells, as shown in panel B. Cells that were negative for all markers are erythrocytes. (C) The total white blood cells per mL calculated by flow cytometry were then compared to the automated CBC WBC count and showed a strong correlation with an r of 0.998, confirming this as an accurate method of cell quantification based on cell surface markers. (D & E) Meg-01 cells were spiked into whole blood and quantified using the flow cytometry protocol. The MK manual count spiked correlated well with the flow cytometry quantified MK count with an r value of 0.980.

**
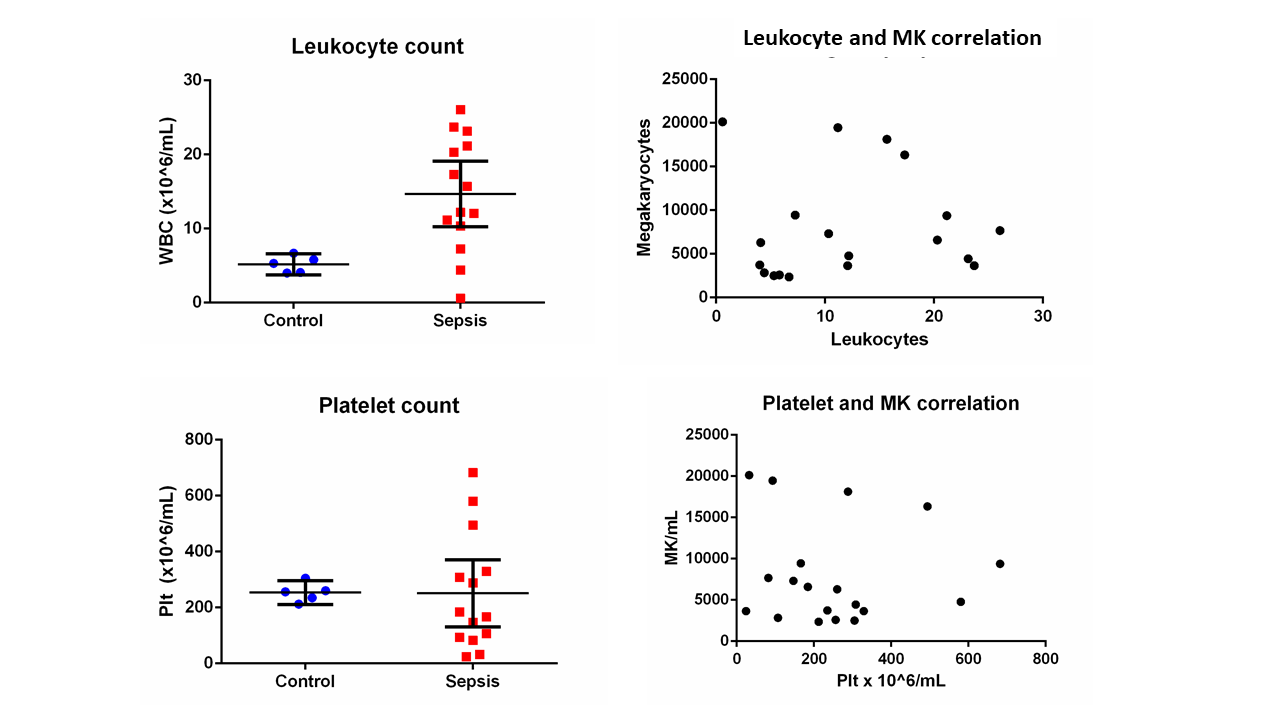
**

**Figure S2. Correlation between CBC and Circulating MKs**

Platelet and white blood cell (WBC; leukocyte) counts were compared to circulating MKs in patients. (A-B) Patients with sepsis had significantly increased WBC counts as compared to controls but there was no significant difference in platelet count. (C-D) There was no correlation between WBC or platelet count and circulating MK count (r2 =0.015 and 0.004, respectively).

**
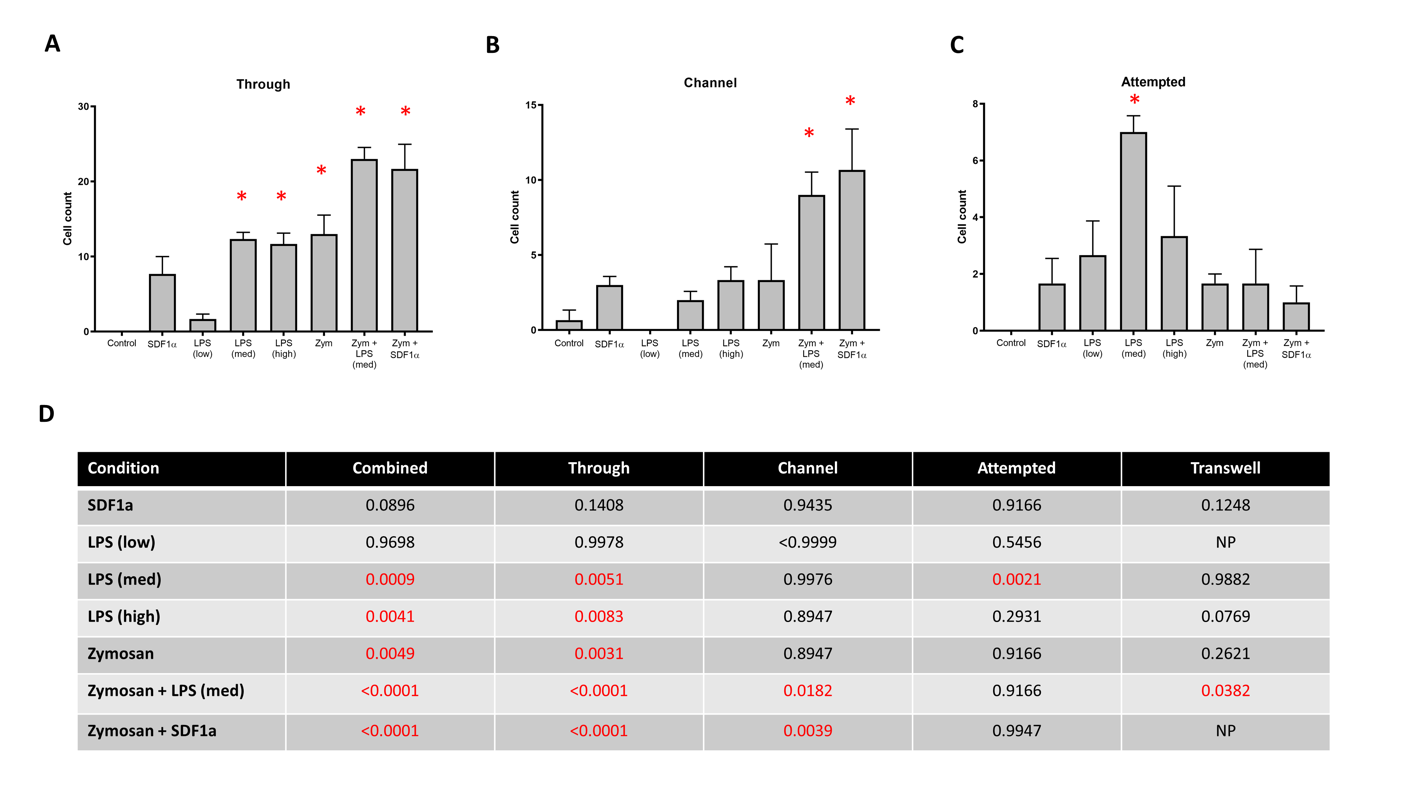
**

**Figure S3. Chemotaxis in microfluidic devices.**

(A-C) Graphs show number of Meg-01 cells scored for different behaviors in microfluidic devices.

D) Table of statistical comparisons of each condition compared to the negative control by one-way ANOVA.

**
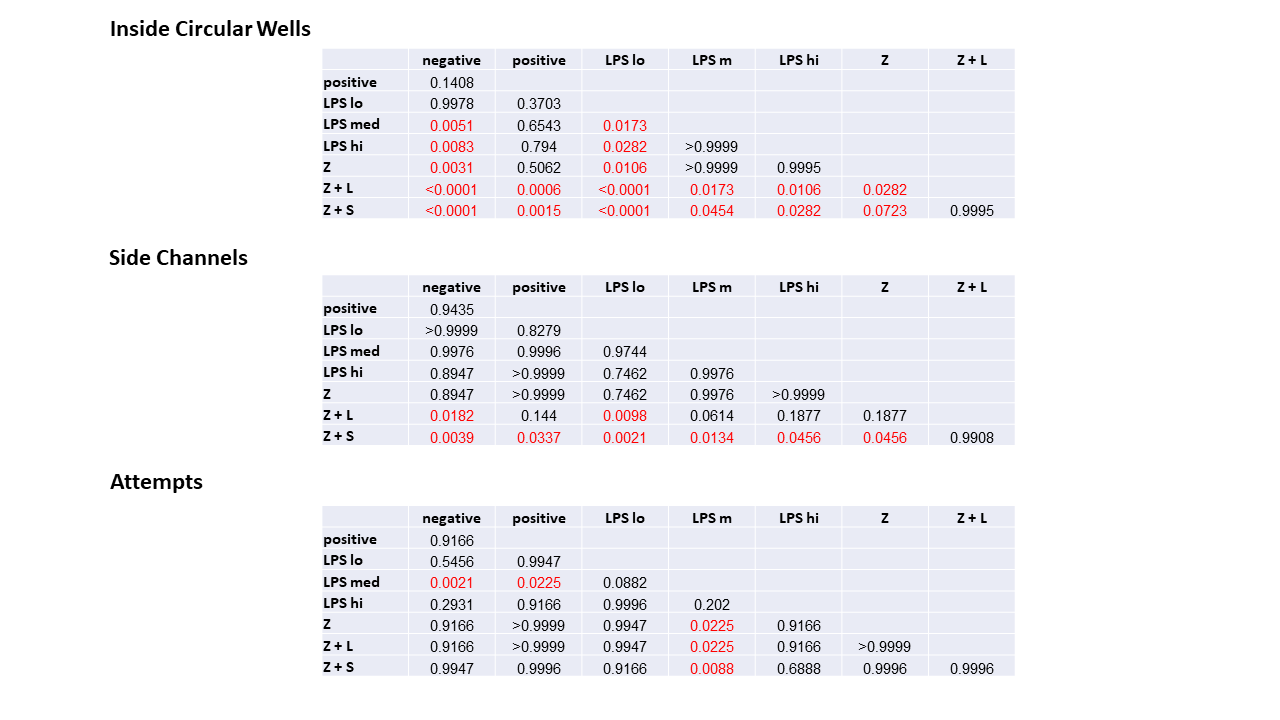
**

**Figure S4. Statistical summary of Meg-01 chemotaxis within a microfluidic device**

One-way ANOVA was performed for each condition shown in Figure 2E. Alpha = 0.05. LPS lo, 22 pg/mL; LPS med, 220 pg/mL; LPS hi, 2.2 ng/mL; Z, zymosan particles; positive, 220 ng/mL SDF1-α; negative, media only; Z, zymosan particles; Z+L, zymosan with 220 pg/mL LPS.

**
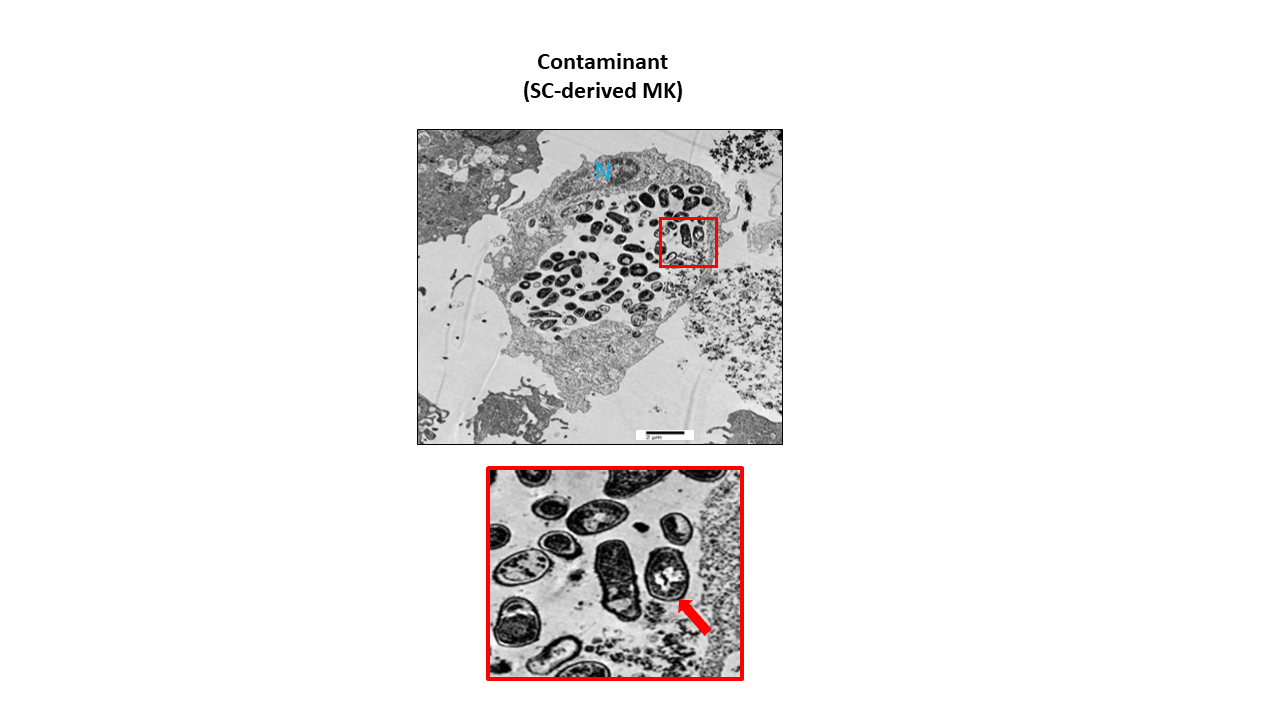
**

**Figure S5: Spontaneous interaction between megakaryocyte and bacteria**Electron Micrographs of an incidental finding of a stem cell-derived MK with multiple bacteria within a large cytoplasmic vacuole from a spontaneously contaminated cell culture.

**
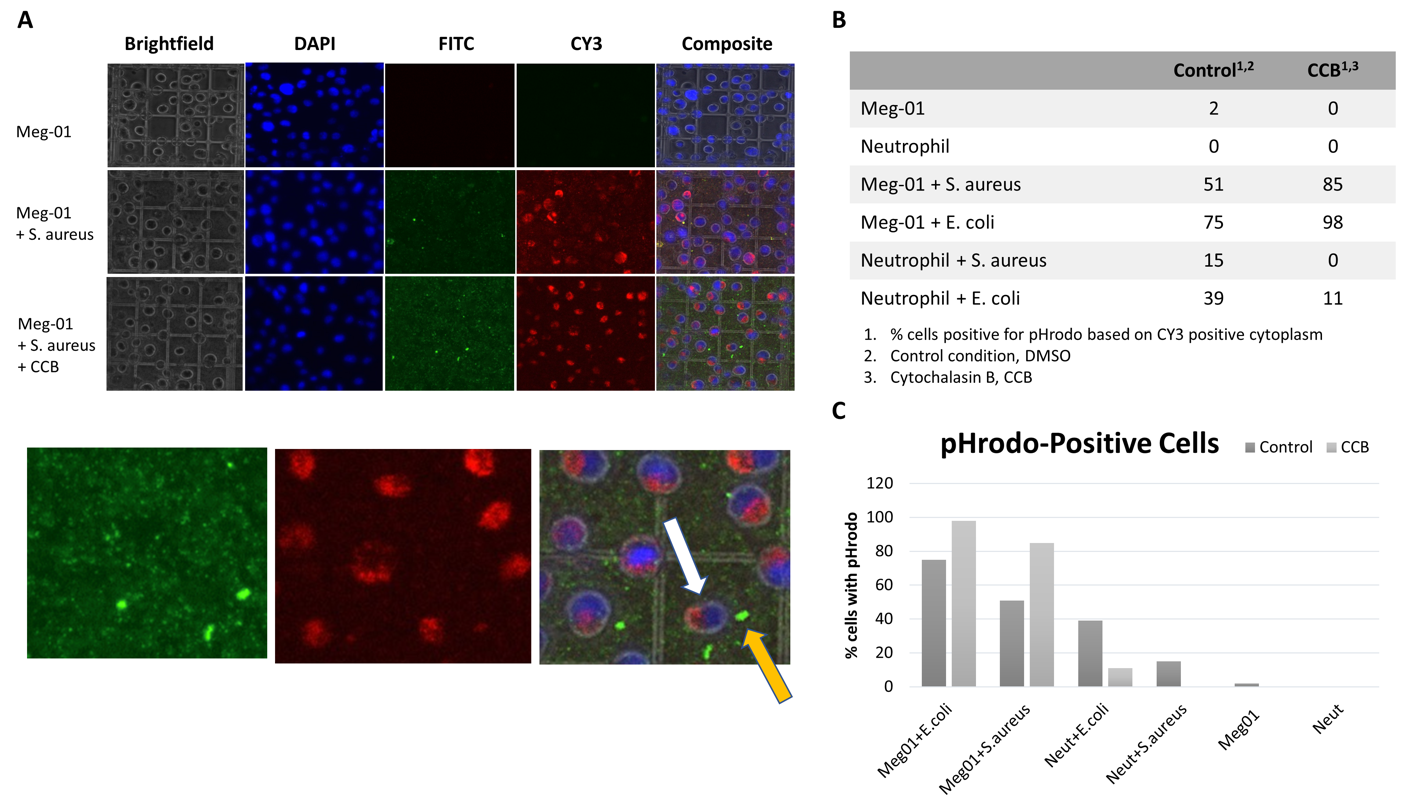
**

**Supplemental Figure 6: Meg-01 cells do not phagocytose *S. aureus* bioparticles.**

A) Shows representative images from phagocytosis experiments, showing that red fluorescence in the cytoplasm of Meg-01 cells is dependent on the presence of *S. aureus* bioparticles labelled with Alexafluor-488 and pHrodo, but is unaffected by the presence of the phagocytosis inhibitor CCB. Magnified images below show that the red fluorescence observed in Meg-01 cells does not correlate with the presence of internalized Alexafluor-488 positive bacterial particles.

B) Quantification of the experiment shown in A with additional data from neutrophils confirming inhibition of *S. aureus* internalization in the presence of CCB.

C) Graph showing a direct comparison of red fluorescence in Meg-01 cells and neutrophils in the presence and absence of CCB. CCB increased red fluorescence in Meg-01 cells and decreased fluorescence in neutrophils.

**
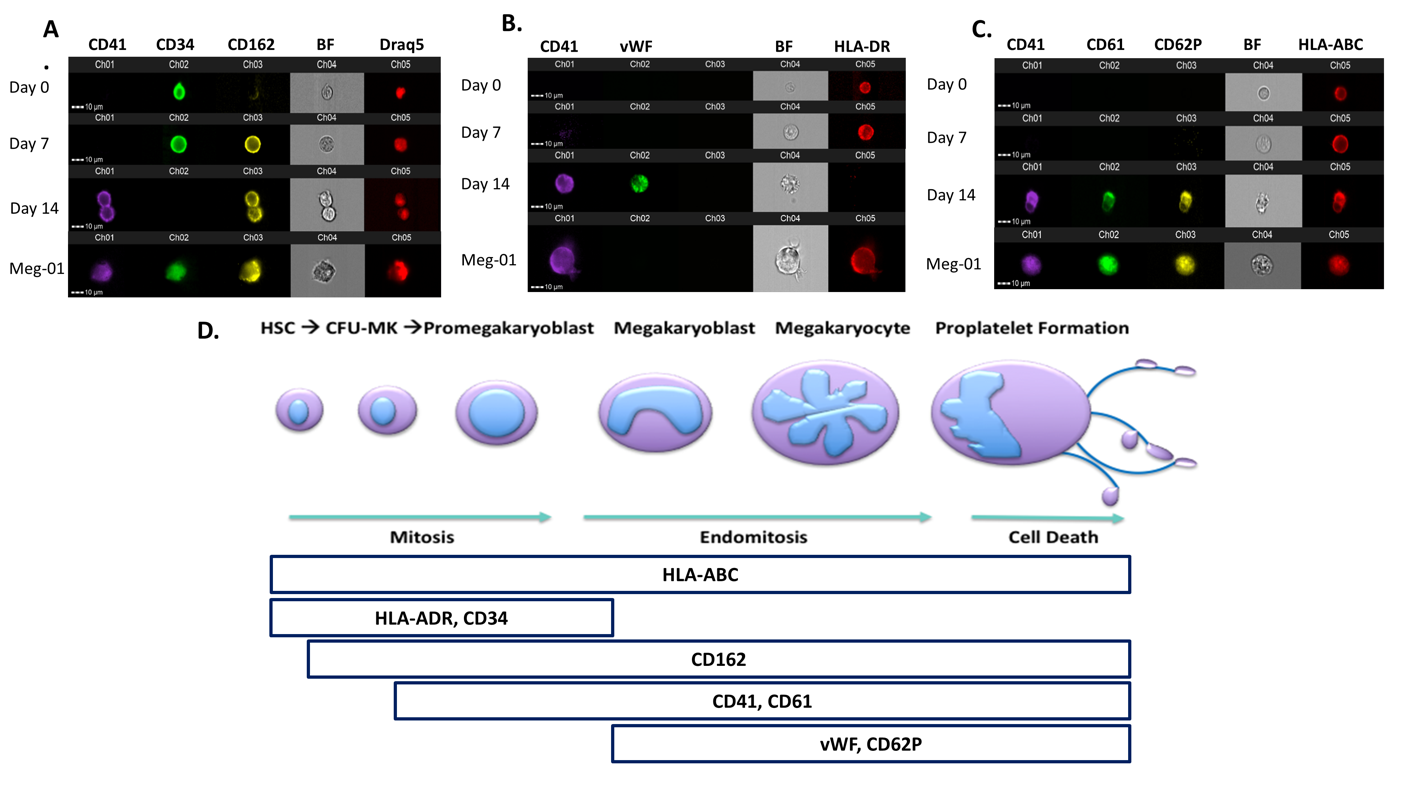
**

**Figure S7: Flow cytometry analysis of cell phenotype.**

Imaging flow cytometry was used to characterize cord blood stem cell--derived MKs (SC MKs) and Meg-01 cells by various cell surface markers, including: CD41 (i-ii), CD34 (i), CD162 (ii), HLA-DR (iii), vWF (iii). HLA-ABC (iv), CD61 (iv), and CD62P Images of representative cells for various days of differentiation (days 0, 7, and 14) are shown in panel G-I. *vWF was not evaluated in Meg-01 cells. Although CD41 is shown to be negative in the day 7 differentiated cells, there was a mixture of CD41^+^ and CD41^-^ cells on day 3 and 7, as shown in Figure S8.


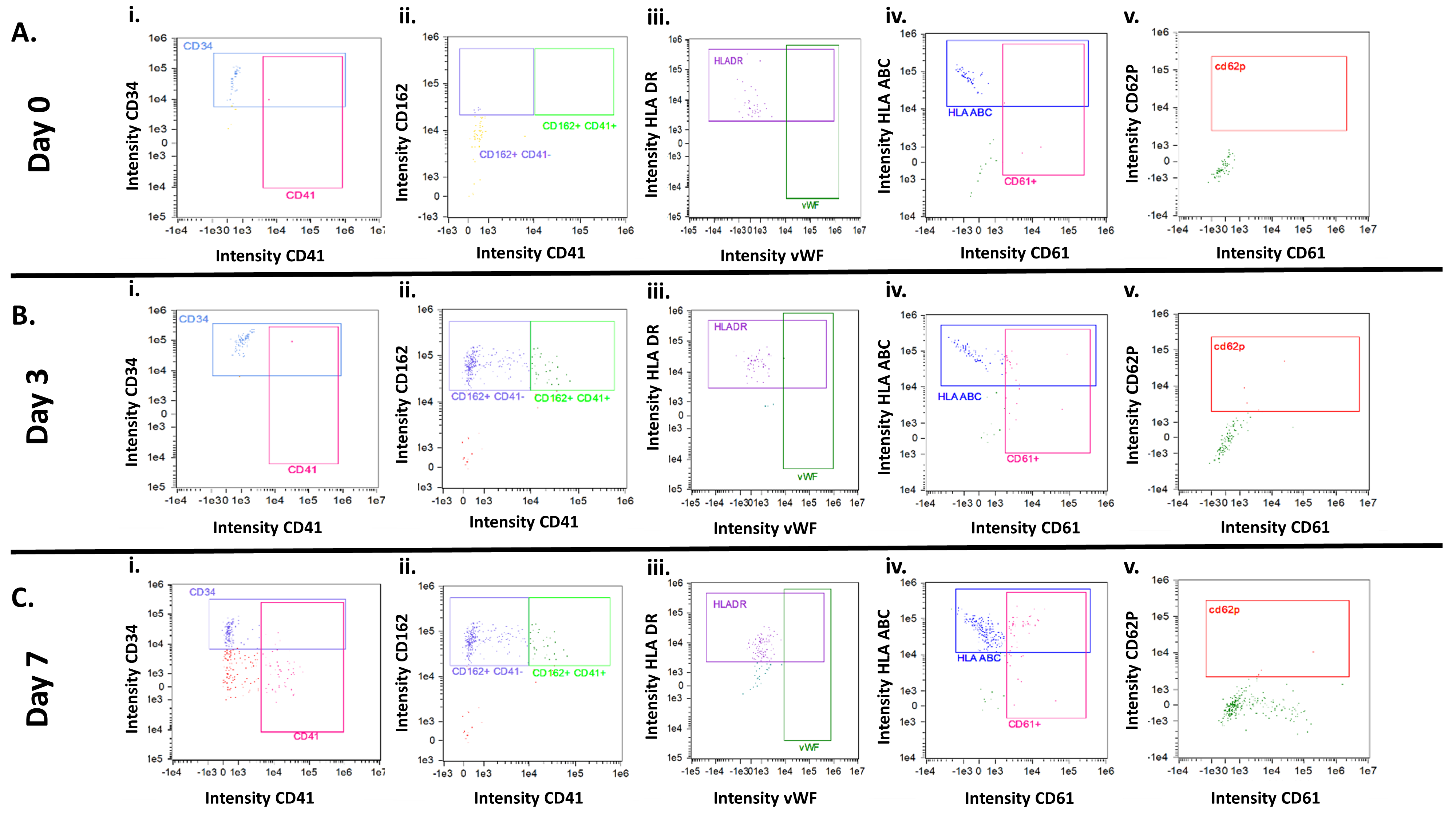


**Figure S8: Flow cytometry analysis of cell phenotype.**

Imaging flow cytometry was used to characterize cord blood SC MKs by various cell surface markers, including: CD41 (i-ii), CD34 (i), CD162 (ii), HLA-DR (iii), vWF (iii). HLA-ABC (iv), CD61 (iv), and CD62P (v). Scatter plots of SC MKs at days 0, 3, 7, 10, and 14 of differentiation are shown in panel A-C.


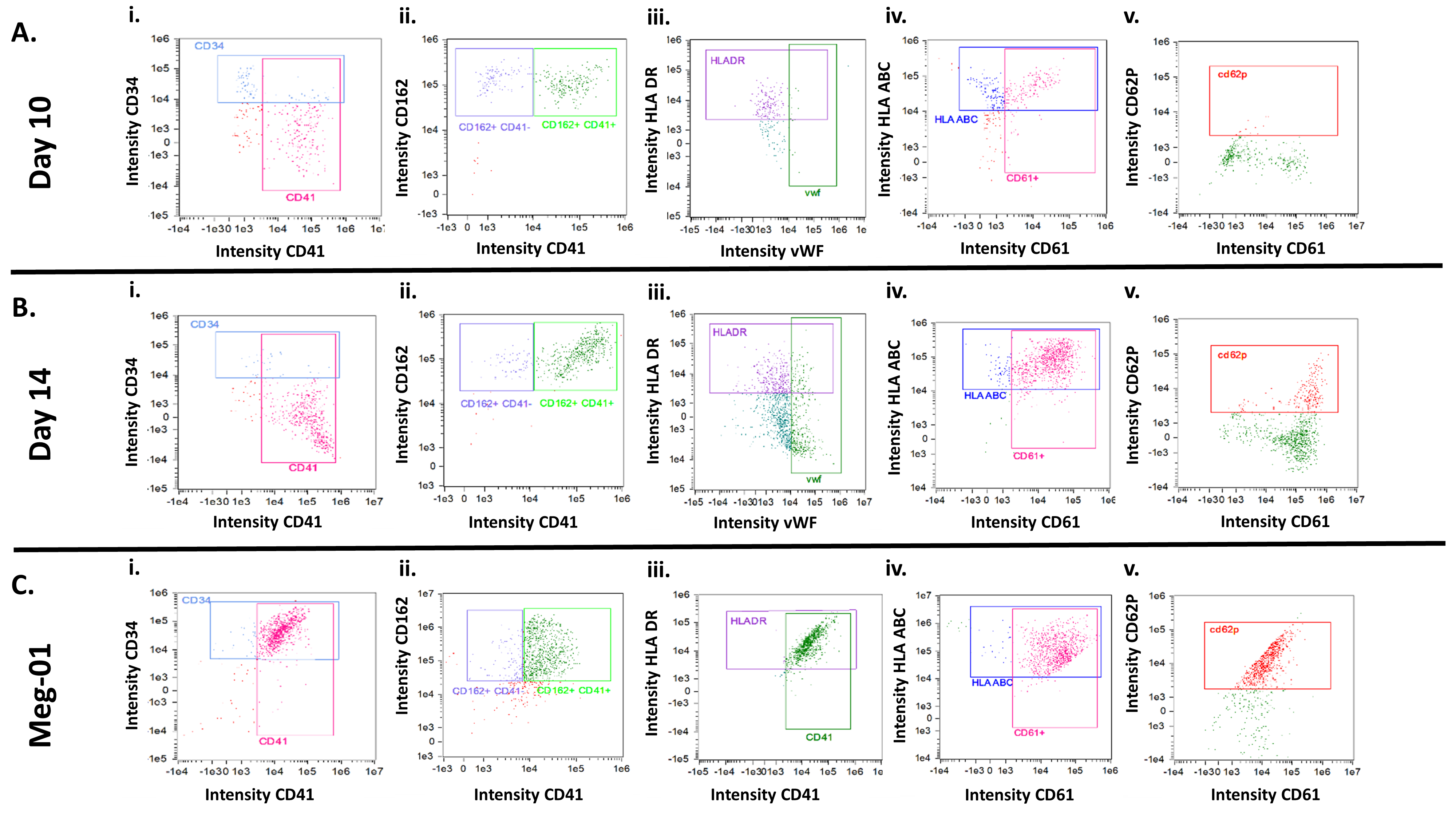


**Figure S9: Flow cytometry analysis of cell phenotype.**

Imaging flow cytometry was used to characterize cord blood SC MKs and Meg-01 cells by various cell surface markers, including: CD41 (i-ii), CD34 (i), CD162 (ii), HLA-DR (iii), vWF (iii). HLA-ABC (iv), CD61 (iv), and CD62P (v). Scatter plots of SC MKs at days 0, 3, 7, 10, and 14 of differentiation are shown in panel A-B and scatter plots of Meg-01 cells are shown in panel C. *vWF was not evaluated in Meg-01 cells, therefore the scatter plot is of CD41 vs HLA-DR (F-iii).

**
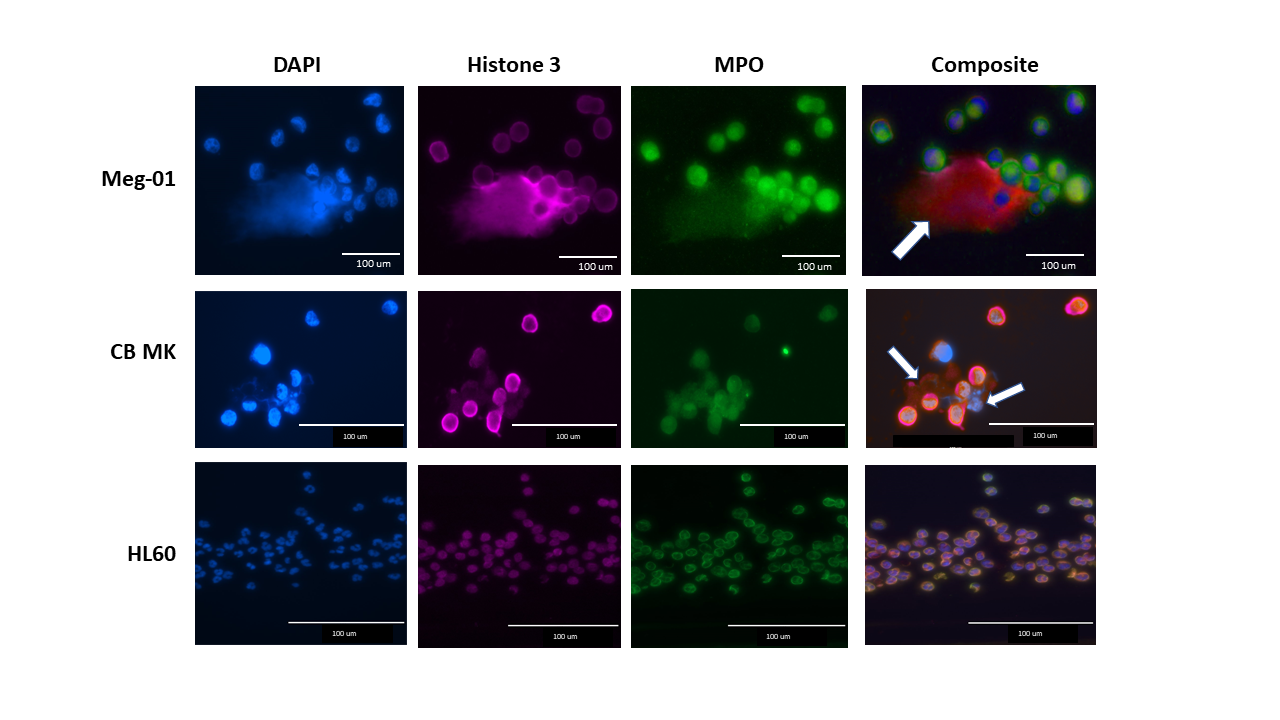
**

**Figure S10:** **MK chromatin webs and histone immunofluorescent staining.**

Immunofluorescence was used to evaluate the chromatin webs associated with Meg-01 and SC MK cells. Unstimulated HL60 cells were used as a control for the immuno-stains. In HL60 cells, DAPI (DNA stain) and Histone 3 (phospho Ser28) staining were both localized to the nucleus of the cells, while myeloperoxidase (MPO) was localized to the cytoplasm. In the Meg-01 cells, the DAPI was localized to the nucleus, the MPO appeared to diffusely stain the cell but brighter staining was present in the cytoplasm of the cell (adjacent to the nucleus), and the Histone 3 staining appeared to form a border around the nucleus, which may indicate prophase. In the Meg-01 and SC MK cells, the chromatin webs stained positive for DAPI, Histone 3, and MPO. White arrow indicates extracellular DNA and histone web location.

**
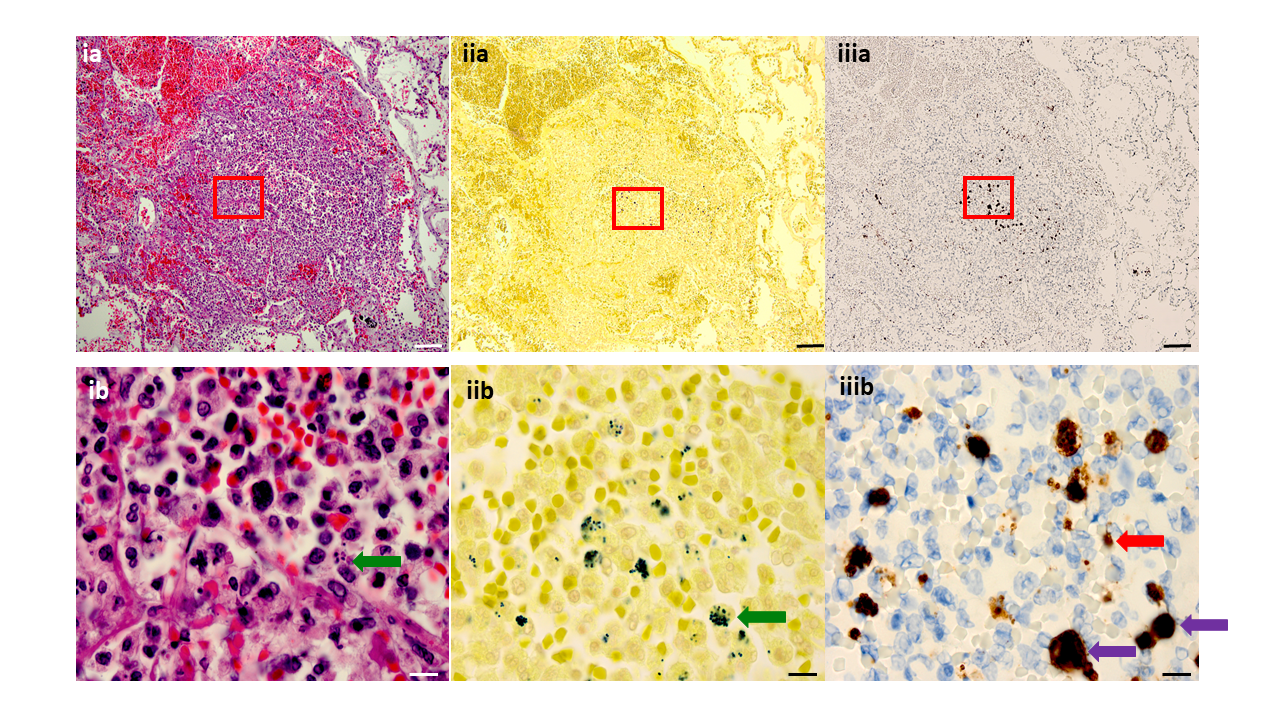
**

**Figure S11: Pulmonary micro abscess in a septic patient**

Histopathological evaluation of a pulmonary abscess in a patient that died from sepsis. Hematoxylin and Eosin (H&E) images of the abscess at 10x (ia) and 100X (iia) shows red blood cells, neutrophils, macrophages, and intracellular cocci (green arrow). Gram stain at 10x (iia) and 100x (iib) showing extra- and intracellular gram positive cocci (green arrow). CD61 staining at 10x (iiia) and 100x (iiib) showing multiple small CD61+ platelets (red arrow) and larger, dark-staining CD61+ cells suspected to be MKs or platelet clusters (purple arrow).

**Supplemental Videos:**

**Video 1: Chemotaxis of Meg-01 cells.**

Meg-01 cells chemotaxing from the main channel into the side comb and side channel into the circular well containing LPS and zymosan particles. Zymosan, red; Nuclear stain (Hoechst), blue. This video was taken over 8 hours.

**Video 2: Platelet-like particle budding within a well after chemotaxis.**

Meg-01 cell that has entered a circular well primed with SDF1-α is observed undergoing apoptosis and budding platelet-like particles within the well. Interestingly, some of the platelet-like particles remain Hoechst-positive, indicating that there is DNA within some of the particles. NOTE: This video was created on a different chemotaxis chamber design, where there is no comb and only one side-channel leading into the well. This video was taken over 8 hours.

**Video 3: Cytoskeletal rearrangement during chemotaxis.**

Continuation of Video 2. Large Meg-01 cell undergoes active cytoskeletal rearrangement with Hoechst-positive granules proximal to the nucleus. The cell is chemotaxing into a circular camber that has SDF1-α and platelet-like particles from a Meg-01 cell that had undergone apoptosis and budded particles. This large cell fills up the entire length of the side channel and then appears to get stuck and stops moving. NOTE: This video was created on a different chemotaxis chamber design, where there is no comb and only one side-channel leading into the well. This video was taken over 8 hours.

**Video 4: Meg-01 cell carrying zymosan particle during chemotaxis.**

Meg-01 cells are observed to chemotax towards wells primed with zymosan particles. One zymosan particle is noted to be within the comb and is carried into the circular well by a cell as it moves into the well. Zymosan, red; Nuclear stain (Hoechst), blue. This video was taken over 8 hours.

**Video 5: Meg-01 cell budding platelet-like particles in the presence of zymosan and LPS.**

Meg-01 cell migrates towards a chamber primed with zymosan and LPS and then proceeds to form connecting platelet-like particles. Zymosan, red; Nuclear stain (Hoechst), blue. This video was taken over 8 hours.

**Video 6: Meg-01 cell budding platelets in the presence of SDF1-α.**

A meg-01 cells is observed to migrate within the side channel, towards a source of SDF1-α in the side chamber. Right before it enters the circular side chamber, the cell stops migration, undergoes cytoskeletal rearrangement, and buds platelet-like particles. The cell does not migrate further once the cytoskeletal changes occurs. Nuclear stain (Hoechst), blue. This video was taken over 8 hours.

**Video 7: Meg-01 cell interacting with bacteria**

Meg-01 cells co-incubated with live *E. coli* bacteria over 4 hours. The cells are noted to migrate throughout the image with some of the cells extending “arms” which appear to contact with multiple of the bacteria. The cells also appear to undergo morphological change as they migrate and interact with the rapidly-multiplying bacteria. Bright-field imaging. Bacteria are seen as small rods which multiply and form clusters. This video was taken over the span of 4 hours.

**Video 8: Bacteria binding to Meg-01 cell**

Meg-01 cells co-incubated with live *E. coli* bacteria overnight. The bacteria are noted to multiple and form clusters. When the bacteria appear to be bound to the cells, they tend to bind to one pole and form clusters around that pole of the cell, while the opposite pole of the cell undergoes some cytoskeletal changes and appears to extend “arms”. In these videos, the bacteria overgrow and appear to overwhelm the Meg-01 cells. This video was taken over the span of 4 hours.
